# Knockin' on pollen's door: live cell imaging of early polarization events in germinating Arabidopsis pollen

**DOI:** 10.3389/fpls.2015.00246

**Published:** 2015-04-21

**Authors:** Frank Vogler, Sebastian S. A. Konrad, Stefanie Sprunck

**Affiliations:** ^1^Cell Biology and Plant Biochemistry, Biochemie-Zentrum RegensburgUniversity of Regensburg, Regensburg Germany; ^2^Faculty of Biology, Institute of Genetics, Ludwig-Maximilians-University of MunichMartinsried, Germany

**Keywords:** pollen activation, cell polarization, bulging, vesicular trafficking, ARO1, actin cytoskeleton, sperm cells

## Abstract

Pollen tubes are an excellent system for studying the cellular dynamics and complex signaling pathways that coordinate polarized tip growth. Although several signaling mechanisms acting in the tip-growing pollen tube have been described, our knowledge on the subcellular and molecular events during pollen germination and growth site selection at the pollen plasma membrane is rather scarce. To simultaneously track germinating pollen from up to 12 genetically different plants we developed an inexpensive and easy mounting technique, suitable for every standard microscope setup. We performed high magnification live-cell imaging during Arabidopsis pollen activation, germination, and the establishment of pollen tube tip growth by using fluorescent marker lines labeling either the pollen cytoplasm, vesicles, the actin cytoskeleton or the sperm cell nuclei and membranes. Our studies revealed distinctive vesicle and F-actin polarization during pollen activation and characteristic growth kinetics during pollen germination and pollen tube formation. Initially, the germinating Arabidopsis pollen tube grows slowly and forms a uniform roundish bulge, followed by a transition phase with vesicles heavily accumulating at the growth site before switching to rapid tip growth. Furthermore, we found the two sperm cells to be transported into the pollen tube after the phase of rapid tip growth has been initiated. The method presented here is suitable to quantitatively study subcellular events during Arabidopsis pollen germination and growth, and for the detailed analysis of pollen mutants with respect to pollen polarization, bulging, or growth site selection at the pollen plasma membrane.

## Introduction

The pollen tube (PT) of flowering plants is formed by the pollen grain vegetative cell and represents a cell of enormous specialization, responsible for the transport of the two male gametes through the female tissues of the pistil to the ovule. It is the fastest elongating plant cell (Sanati Nezhad et al., 2014) and can reach lengths of 30 cm, with growth rates up to 1 cm/h (Mascarenhas, 1993). PT growth is monotropic by expansion at an annular region located at the tip in a process called polar tip growth (Taylor and Hepler, 1997; Geitmann, 2010).

Deeply embedded in the tissues of the pistil, *in vivo* PT growth is difficult to investigate with high temporal and spatial resolution and has been achieved so far only by using two-photon microscopy (Feijó and Moreno, 2004; Cheung et al., 2010). As an advantageous alternative, pollen can be germinated *in vitro* to study the cellular dynamics and complex signaling pathways that coordinate polar tip growth (Qin and Yang, 2011). From these studies we know that intensive exo- and endocytosis at the tip supported by regulated vesicle trafficking and cytoskeleton dynamics, as well as coordinated changes in cell wall properties are essential cellular activities of the growing PT (for review see Geitmann, 2010; Guan et al., 2013). Great advances have been made during the past years in identifying key signaling molecules for the proper elongation of the PT tip, such as Rho GTPases, calcium ions, and phosphoinositides (for review see Cheung and Wu, 2008; Qin and Yang, 2011; Steinhorst and Kudla, 2013). These key regulators are components of distinct signaling pathways forming a complex network that controls the cellular activities of tip-growing PTs (Guan et al., 2013). However, there are still significant gaps in our knowledge of PT growth regulation, especially with regard to the question when and how symmetry breaking in the apparently unpolar pollen vegetative cell occurs, and what the molecular mechanism for selecting the growth site is.

Polar tip growth of PTs is very similar to the polar elongation of root hairs on genetic and mechanistic levels (reviewed in Šamaj et al., 2006; Campanoni and Blatt, 2007; Kost, 2008; Lee and Yang, 2008). Root hair growth is known as a multi-phasic process, consisting of cell fate determination, the formation of a root hair bulge, and the initiation of tip growth in the root hair bulge, each of which is characterized by distinct physiological and mutant phenotypes in the model plant Arabidopsis (Schiefelbein and Somerville, 1990; Parker et al., 2000; Schiefelbein, 2000; Bibikova and Gilroy, 2003; Müller and Schmidt, 2004). Since pollen germination and the initiation of PT tip growth is rapid and much faster than root hair growth, it is technically more demanding to perform live cell imaging in order to study the cellular dynamics and the growth kinetics during pollen hydration, activation, germination and PT formation. Moreover, *in vitro* germination rates and growth dynamics of Arabidopsis pollen are known to be highly variable (Johnson-Brousseau and McCormick, 2004; Boavida and McCormick, 2007), complicating its use for cellular and molecular genetic studies of pollen germination and growth. However, methodological advances in germination techniques meanwhile facilitated the experimental use of Arabidopsis pollen (Bou Daher et al., 2009; Rodriguez-Enriquez et al., 2013; Vogler et al., 2014), offering possibilities to establish methods for larger-scale screening and quantitative phenotyping of wild type and mutant pollen.

To optimize high throughput time-lapse live imaging of germinating Arabidopsis pollen, we established an inexpensive and easy mounting technique suitable for every standard microscope, based on an improved pollen germination medium (Vogler et al., 2014). Using this setup for Spinning Disc confocal microscopy we investigated the growth kinetics and morphology changes of Arabidopsis PTs expressing GFP in the cytoplasm of the vegetative pollen cell. We focused on early cell polarization events during pollen activation and germination by studying the spatiotemporal localization of GFP-labeled Armadillo Repeat Only 1 (ARO1), which is known to be essential for polar PT growth (Gebert et al., 2008). ARO1-GFP accumulates in the inverted cone-shaped region of growing PT tips in a brefeldin A and latrunculin B sensitive manner and TIRF microscopy, applied in this study, confirmed that ARO1-GFP localizes vesicle-associated at the PT tip.

We used Arabidopsis marker lines expressing ARO1-GFP and tagRFP-T-Lifeact in the pollen to study vesicle and filamentous actin (F-actin) dynamics before and during pollen germination. Furthermore, we used a pollen marker line with fluorescently labeled sperm cell nuclei and plasma membranes (Sprunck et al., 2012) to address the question when the two sperm cells, physically linked to the nucleus of the vegetative cell forming a male germ unit (MGU) (McCue et al., 2011; Zhou and Meier, 2014), are transported from the pollen grain into the germinated PT.

Our time-lapse live imaging of germinating Arabidopsis PTs revealed similarities between root hair formation and pollen germination as we observed successive phases of cell polarization, bulge formation, growth site selection, and the initiation of rapid tip growth. Prior to pollen germination, we observed a characteristic polarization of vesicle-associated ARO1-GFP and tagRFP-T-Lifeact labeled F-actin in the pollen grain. After bulging, a transition phase is observed where vesicle-associated ARO1-GFP heavily accumulates at the distal end of the bulge and adopts an inverted cone-like shape before the PT switches to rapid tip growth. At the same time, long F-actin cables appear, extending in parallel orientation from within the pollen grain into the PT, while the volume of the vacuole, arising opposite the germination site, increases. During the phase of rapid tip growth, F-actin bundles massively accumulate at the germination site and increasing vacuolization occurs, followed by sperm cell transport into the PT.

## Materials and methods

### Plant material

*Arabidopsis thaliana* (accession Col-0) plants were grown under long-day conditions (16 h light, 20°C, 70% humidity) in growth chambers after seeds were subjected to stratification 2 days at 4°C in the dark. Homozygous lines carrying the *P_*Lat*52_:GFP* transgene were used to express cytoplasmic GFP in the vegetative PT cell (Twell et al., 1990). A C-terminal GFP fusion of the ARO1 protein under control of its endogenous promoter (Gebert et al., 2008) was used to investigate its subcellular localization in pollen and PTs. A double homozygous marker for *P_*HTR*10_:HTR10-RFP* and *P_*HTR*10_:TET9-GFP* line (Sprunck et al., 2012) was used to visualize sperm cell nuclei and sperm cell plasma membranes.

### Molecular cloning and generation of transgenic lines

A double stranded DNA fragment encoding for a 17 aa actin binding domain termed Lifeact (Riedl et al., 2008) with additional 5′ and 3′ *Hind*III restriction sites was synthesized by proof-reading PCR on the partially overlapping template oligonucleotides 5′-GGGGCCATGGAAGCTTTGGGACCAGC CGTAGGAATGGGTGTTGCTGATCTTATTAAGAAGTTCGA GTCTATTTCTAAGGAGG-3′ and 5′-GGGGAAGCTTATGCC ATGGCTCCAGCTACAGGTGCTCCCGCCCCTCCTTCCTCC TTAGAAATAGACTCGAACTTCTTAA-3′ with the PCR primers Lifeact-fwd (5′-GGGGCCATGGAAGCTTTGG-3′) and Lifeact-rev (5′-GGGGAAGCTTATGCCATGGC-3′). After *Hind*III digestion, the PCR product was ligated behind the fluorophore coding sequence into the modified Gateway destination vector pENTR-tagRFP-T (Denninger et al., 2014) to obtain pENTR-tagRFP-T-Lifeact. To achieve expression in pollen, 712 bp of the *ARO1* promoter with additional 5′ *Sac*I and 3′ *Spe*I sites were amplified from the 95P-Nos-ARO1p:ARO1-GFP plasmid (Gebert et al., 2008) with the primers pARO1-II-for (5′-TCGGGTACCGAGCTCAGATCTAAGCTG-3′) and pARO1-II-rev (5′-TGTCGACGCGGCCGCACTAGtCAGATC-3′). The *35S* promoter of the binary gateway expression vector pB2GW7 (Karimi et al., 2002) was replaced by the *ARO1* promoter via *Sac*I/*Spe*I to obtain pB2GW7-ARO1p. Gateway LR reaction with pENTR-tagRFP-T-Lifeact and pB2GW7-ARO1p was performed according to the manufacturer's recommendations (Life Technologies) to obtain the expression vector pARO1:tagRFP-T-Lifeact that was used for *Agrobacterium*-mediated plant transformation by floral dip method (Clough and Bent, 1998).

### Pollen mounting and live cell imaging

Micro-germination slides were prepared in either a single-well or a multi-well setup (Figures S1,S2). To prepare a single-well micro-germination slide, a 1–2 mm high planar plasticine layer was added on the margin of the ring of a slide with an attached glass ring (L4246, PLANO, Wetzlar, Germany). The well was filled with molten pollen germination medium (PGM) according to Vogler et al. (2014), containing 10 μM 24-epibrassinolide (epiBL, Sigma-Aldrich E-1641) and solidified with 0.5% low melting point agarose. After solidification the center of the well was hand pollinated using single dehiscent anthers, manually removed from flowers at flower stage 13–14 (according to Smyth et al., 1990). The well was then sealed by gently pressing evenly a 24 × 24 mm No. 1.5 cover slip onto the plasticine until it slightly touched the PGM. The illustrated instruction on how to prepare a single-well micro-germination slide is shown in Figure S1. Multi-well micro-germination slides were prepared by attaching a 12 well silicon profile (flexiPERM® micro12, SARSTEDT, Germany) to a standard microscope slide (26 × 76 mm) and filling each well with 50–75 μL molten PGM. After solidification, the silicon profile was removed and another 25 μL of molten PGM were added on top of each agar pad to obtain convex shapes. After a frame of plasticine was modeled around the agar pads, they were hand pollinated and then sealed by gently pressing evenly a 24 × 60 mm No. 1.5 cover slip on the plasticine frame. The scheme on how to prepare a multi-well micro-germination slide is shown in Figure S2. Immediately after pollen application, micro-germination slides were used for live-cell imaging. No obvious differences in germination or PT growth were observed between single-well or multi-well micro-germination setups. By contrast, much lower and highly variable germination rates as well as slower PT growth rates were observed when PGM without 10 μM epiBL was used to prepare the micro-germination slides, while the different phases of pollen germination and tube growth described in this work were unaffected.

Microscopy was performed on a ZEISS Cell Observer Spinning Disc confocal microscope (Yokogawa CSU-X1) equipped with a motorized stage using 20×/0.8 NA dry, 40×/1.30 NA DIC oil immersion or 63×/1.40 NA DIC oil immersion objectives. GFP fluorescence was excited with a 488 nm laser line and emission was detected from 505 to 545 nm. A 561 nm laser line was used to excite tagRFP-T and emission was detected from 570 to 640 nm. Free GFP in the pollen cytoplasm and ARO1-GFP fusion protein were imaged every 3 min, tagRFP-T-Lifeact every 10–15 min over 4–6 h in z-stacks of 11 optical slices at each of 10–20 positions representing individual pollen spots.

### Morphological modeling of pollen germination

We assumed two extreme morphological models describing cellular geometries of germinating PTs and simulated these models graphically with Illustrator CS4 software (Adobe). In the “protrusion model” we proposed linear growth at the tip of a protuberance, generating a constantly elongating cylinder with a dome-shaped tip that emerges from the germination site. In the “bulging model” a first phase of isodiametric inflation was assumed for the germinating PT, followed by a second phase in which isodiametric growth switches to polar growth at a dome-shaped tip. Thus, in the “protrusion model,” a tubular object constantly emerges out of an ellipse, representing the pollen grain. To generate the “bulging model” a circle, representing the PT, was placed below the upper margin of an ellipse, representing the pollen grain. The diameter of the circle was frame-wise and constantly increased, while keeping its position constant at the lowermost point. After 20 frames, we changed the distal region of the circle into a dome-shaped tip, which then constantly elongates in form of a cylinder like in the “protrusion model.” In both morphological models, the width of the dome-shaped tip was set identical and did not change during elongation. Furthermore, the net increase in PT area was set identical for both models. Modeled PTs were measured in ImageJ like described for microscopic images (Image Processing and Quantitative Analysis).

### Pollen staining and microscopy

For membrane staining with FM4-64, pollen of ARO1-GFP expressing plants were germinated in 35 mm petri dishes on solidified PGM as described above. Three hours after pollination a small agar piece was excised and mounted upside down on a cover slip in a droplet of 8 μM FM4-64 (Life Technologies) dissolved in liquid PGM. Images were taken with an inverted SP8 Confocal Laser Scanning Microscope (Leica Microsystems) with a 40×/1.3NA oil immersion objective and 1 airy unit pinhole opening. GFP and FM4-64 were excited simultaneously with a 488 nm laser line. GFP emission was detected from 495 to 550 nm and FM4-64 emission from 650 to 725 nm using HyD detectors. For DAPI staining, pollen of plants expressing *P_*ARO*1_:tagRFP-T-Lifeact* were put in a droplet of DAPI staining solution (2.5 μg/ml 4′,6-diamidino-2-phenylindole (DAPI), 0.01% Tween-20, 5% DMSO, 50 mM PBS, pH 7.2). Confocal z-stacks were acquired at the Spinning Disc system described above using a 100×/1.40 NA oil immersion objective. DAPI fluorescence was excited with a 365 nm LED illumination (COLIBRI, ZEISS) and emission light was filtered by the microscope stand built-in filter cube (emission filter: 447–507 nm) and channeled through an empty Spinning Disc position to display DAPI fluorescence on the same camera as for tagRFP-T and DIC channels.

### TIRF microscopy

For TIRF microscopy of PTs, a very planar gel pad was generated by laying two microscope slides orthogonal on the edges of three adjacent slides (Figure S3). 500 μl of molten PGM containing 2% agarose was pipetted to the middle of the lower slides and immediately covered with another slide. After solidification, the uppermost slide and all flanking slides were removed and the PGM pad was hand pollinated as described above (Pollen Mounting and Live Cell Imaging). Pollinated slides were kept in a damp box for 3–5 h. Prior to microscopy, a droplet of double distilled water was pipetted onto the pad and a No. 1.5H cover slip was added. TIRF illumination was generated in a Delta Vision Elite (GE, Healthcare, Applied Precision) system with an Olympus IX-71 microscope, equipped with an Insight SSI(TM) solid state illumination system and an X4 laser module. Images were taken with an Olympus UAPON 100XOTIRF 1.49 NA oil immersion objective and recorded with a CoolSnap HQ2 CCD camera (Photometrics, Tucson, USA). GFP was excited with the 488 nm laser line and emission was detected between 501 and 549 nm. Image exposure time and TIRF angle were adjusted according to sample fluorescence intensity and specimen location.

### Image processing and quantitative analysis

All images were processed in ImageJ (http://rsbweb.nih.gov/ij, version 1.45). In time-lapse experiments, the frame before a PT emerged from the germination site was set to zero. Z-stacks of time lapse images of pollen expressing *P_*Lat*52_:GFP* were subjected to projection algorithms. Bright field images were sum slice, GFP images maximum intensity projected. Afterwards, for the GFP channel a threshold was applied to obtain binary images. The implemented WAND tool was used to determine the pollen area, which was then subtracted from all images for a given PT and subsequently the PT area was measured for each frame. The analysis of PTs was only carried out with those PTs where growth was not disturbed by any other object and which could be observed for at least 1 h. Of all PTs the shape descriptor “roundness” was measured, given by 4^*^area/(pi^*^(major axis)^2^) of a respective PT. To compare the frame wise PT area increase shortly after germination and in later PT growth phases, the mean frame wise increase of the first 10 and the last 10 frames was calculated and compared in a Friedman's 2-way variance analysis. Z-stacks of time lapse images of pollen expressing *P_*ARO*1_:ARO1-GFP* were also subjected to maximum intensity projections first. For those pollen that were monitored at least half an hour before and after the time point of germination, images were cropped in a rectangular selection containing only the pollen and emerging PT. All frames of a PT were included in a stack histogram that was used for subsequently computing gray values for setting a 70% signal threshold to determine the PT shape (false colored in red) and a 0.5% signal threshold (false colored in yellow) to determine the maximum intensity peaks for ARO1-GFP. Frame-wise PT area increase was measured by overlaying unbiased PT shape outlines that were obtained using the WAND tool, which was also used to determine the size of individual PT areas. ARO1-GFP maximum intensity peaks were quantified using a variable ROI selection and measuring its mean gray value that was subsequently multiplied by the ROI size. To compare multiple PTs in a mean value computation, ARO1-GFP maximum intensity was normalized for each PT to its maximum signal value. Z-stacks of images of pollen expressing tagRFP-T-Lifeact were maximum intensity projected and to better visualize the maximum signal intensities false colored using the “spectrum” LUT. Calculations were performed with Excel2010 (Microsoft) and statistical analyses were computed with SPSS22 (IBM).

## Results

### Live cell imaging of pollen germination and PT growth

**Figure 1:**
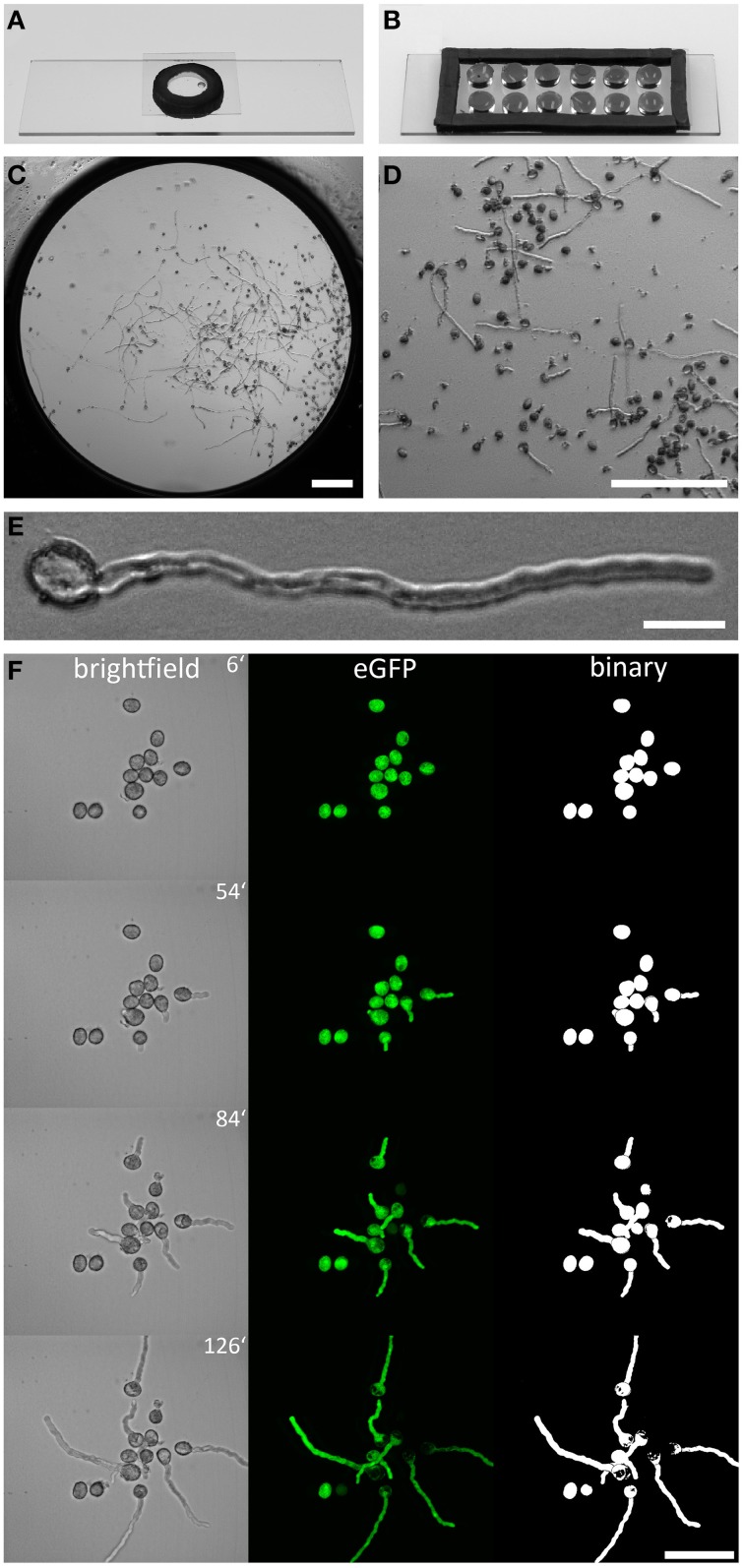
**Arabidopsis pollen imaging in the micro-germination setup**. Mounting pollen in a single **(A)**, or multi well **(B)**, micro-germination setup for live cell imaging. In the center of one well **(C)** PTs grow in close proximity to the cover slip **(D)**, allowing the use of high NA immersion objectives with low working-distances. Pollen germination is not affected by the mounting technique and PTs showed normal morphology **(E)**. A time series of germinating *P_*Lat*52_:GFP* pollen is shown in **(F)**. Brightfield images are shown as sum slice projections and confocal fluorescence images of cytoplasmic GFP as maximum intensity projections. Binary images of GFP channel enable unbiased quantitative measurement of PT area size and morphology **(F)**. Scale bars: **(C,D)** 200 μm, **(E)** 20 μm, **(F)** 50 μm.

To facilitate time lapse live cell imaging of Arabidopsis pollen germination and PT growth using high NA immersion objectives, we designed a single-well and a multi-well micro-germination setup as shown in Figures 1A,B. Both setups are fast and easy to prepare (see Figures S1,S2), based on inexpensive components. Pollen germination and PT growth of up to 12 genetically different plants can be simultaneously observed over many hours when using the 12-well micro-germination setup. Pollen germinates in the direct proximity to the cover slip in a film of PGM that is formed when the cover slip is gently pressed on the medium to seal the well (Figure 1C). Pollen germination rates within this film are very high (>80%) and homogenous (Figures 1D,F), with normal PT morphology (Figure 1E). In an exemplary 10 well-setup, no temporal or morphological deviations in pollen germination or PT growth were observed (Supplemental Movie 1). This technique can be broadly used in every lab, adapted to many microscopic techniques and may be even up-scaled for the simultaneous imaging of pollen from more than 12 individuals.

### Pollen tube growth kinetics

**Figure 2:**
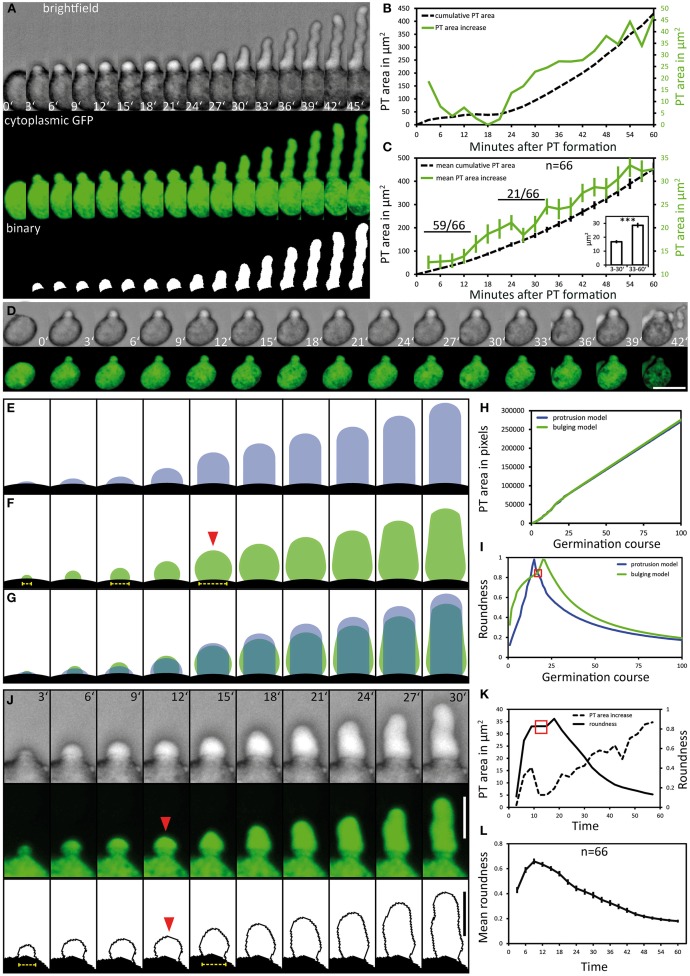
**Pollen tube growth kinetics and morphology changes**. Pollen expressing GFP in the cytoplasm of the vegetative cell (*P_*Lat*52_:GFP*) was used to quantitatively assess the kinetics of PT growth and PT morphology during germination. Brightfield and fluorescence channel, together with the binary image generated from the fluorescence channel, are shown in **(A)**. Time point 0′ indicates the last frame before germination. The quantification of the cumulative PT area and frame-wise increase in PT areas over time is given in **(B)**. Mean values ± 1 SE of cumulative PT areas and the frame-wise increase in PT areas of 66 evaluated PT's are shown in **(C)**. Numbers in **(C)** represent the frequency of an observed pattern. The mean frame-wise increase in PT areas for the first 10 frames of each pollen tube is significantly lower compared to the last 10 frames of each PT [inset of **(C)**; asterisks indicate statistically highly significant differences, *p* < 0.001]. A pollen that germinates but fails to bursts after germination is shown in **(D)**. “Protrusion” and “bulging” model for PT morphology changes during pollen germination and PT elongation are shown in **(E,F)**. The overlay of both morphological models is shown in **(G)**, their PT area increase over time is shown in **(H)**. The course of “roundness” for both morphological models is shown in **(I)**. Red solid arrowhead in **(F)** and red square in **(I)** highlights the transition from unpolar bulging to polar elongation in the “bulging model.” A representative germinating PT is shown in **(J)** and its course of PT area increase and “roundness” is shown in **(K)**. Mean values ± 1 SE of the “roundness” of 66 PTs are given in **(L)**. Scale bars: **(A,B)** 25 μm, **(J)** 12.5 μm.

We evaluated a total of 66 PTs expressing cytoplasmic GFP in the PT vegetative cell that fulfilled our quality criteria for quantitative PT analyses, that is the absence of any obstacle during germination and growth and the complete recording of at least 1 h after germination. As PTs represent 3-dimensional cylindrical objects, we did not determine PT length in μm but measured the PT as area in μm^2^. Automatic size measurements using the WAND tool (ImageJ) were performed with thresholded binary images of maximum intensity projections (Figure 2A). The growth kinetics of this PT is depicted as frame-wise increase in PT area and as cumulative increase in PT area over time, respectively (Figure 2B). After germination, no marked increase in PT area can be observed during the first 21 min of PT growth. Twenty four minutes after germination, PT growth strongly increases and rises even more after 42 min. Comparing the growth rate determined by PT area measurements with PT length as a measure of growth revealed similar growth kinetics (Figure S4). When we estimated the ratio of PT length to PT area over time, we calculated an approximated conversion factor of 0.18 μm^−1^ for transferring PT area (μm^2^) in PT length (μm).

When all 66 PTs were included into the quantification of growth kinetics, a high overall PT growth rate of 400.2 μm^2^/h (mean cumulative PT area) was observed (Figure 2C). We observed no or only very low increase in the PT area during the first 12 min of PT formation, resembling a lag phase. To statistically test this, we compared the mean PT area increase rate for the first and the last 10 frames of PT growth (inset in Figure 2C). In almost all cases (59 of 66), the mean PT area increase during the first 30 min was lower than for the last 30 min. Friedman's 2-way variance analysis revealed that PT growth during the first 30 min is highly significantly slower (*p* < 0.001) than during the last 30 min.

From these results we conclude that PT growth in Arabidopsis starts with a first distinct phase of slow growth shortly after germination that is followed by a second phase of rapid PT growth. This is furthermore corroborated by the finding that in case of PT burst, also a short protuberance is first initiated but obviously does not pass over to the next phase of rapid elongation (Figure 2D). A later short phase of decelerated growth could be observed in about one third (21 of 66) of all PTs investigated (Figure 2B). This lag phase occurred when PTs reached a mean size of approximately 200 μm^2^ and it was more variable and less pronounced (Figure 2B; Figure S5).

### Changes in PT shape after pollen germination

We investigated the PT morphologies during germination and the transition to rapid tip growth in more detail and compared them with two extreme morphological models describing possible PT geometries. The “protrusion model” assumes that a PT would emerge from the pollen grain as an elongating cylinder with a dome-shaped tip that is maintained during germination and rapid tip growth (Figure 2E). As a result the diameter of the junction between the pollen grain and the PT, which is the site of germination, would remain rather constant in this model. By contrast the “bulging model” assumes that the PT initially exhibits isodiametric growth, leading to a round bulge emerging at the germination site (Figure 2F, red arrowhead). In a second phase, isodiametric growth would have to switch to polar tip growth by selecting a growth site and forming an elongating cylinder. A unique feature of the “bulging model” is that the isodiametric inflation of the bulge will increase the diameter of the germination site over time.

The differences between the two morphological models are highlighted in Figure 2G. For both models we determined a similar net increase in PT area and the same width of the dome-shaped tip. When we compared the increase in PT area over time it was indeed almost identical for both models (Figure 2H). We computed the course of “roundness” for both morphological models during germination and found the “roundness” to increase linearly to a sharp peak in the “protrusion model,” followed by a rapid decrease when the PT continues to elongate (Figure 2I). In the “bulging model,” by contrast, the course of roundness of a germinating PT forms a rather hyperbolic increasing curve with a broader maximum leading to an accentuated peak (Figure 2I).

Notably, live imaging of germinating pollen revealed considerable similarities to the “bulging model” (Figures 2J,K). During the first 12 min of germination the pollen vegetative cell forms a round bulge at the germination site that exhibits isodiametric growth. Afterwards, the uniformly expanding bulge undergoes the transition into a polar growing PT (Figure 2J; 15′ to 18′). The changes in its shape are reflected by the course of “roundness” plotted for this PT (Figure 2K). A hyperbolic increase with a broad maximum (red box in Figure 2K) is characteristic for the phase of bulging. The following peak defines the transition phase, when the bulge starts to form a dome-shaped tip, followed by a switch to rapid tip growth (Figure 2K).

We found the same tendency when we plotted the mean course of “roundness” for all 66 PTs (Figure 2L). Furthermore, in 37 of 66 examined pollen the increasing diameter of the germination site during bulging was clearly visible (Figure 2J, yellow dotted line), which is in line with the unique feature predicted by the “bulging model” (Figure 2F, yellow dotted line).

### ARO1-GFP is associated to vesicles

**Figure 3:**
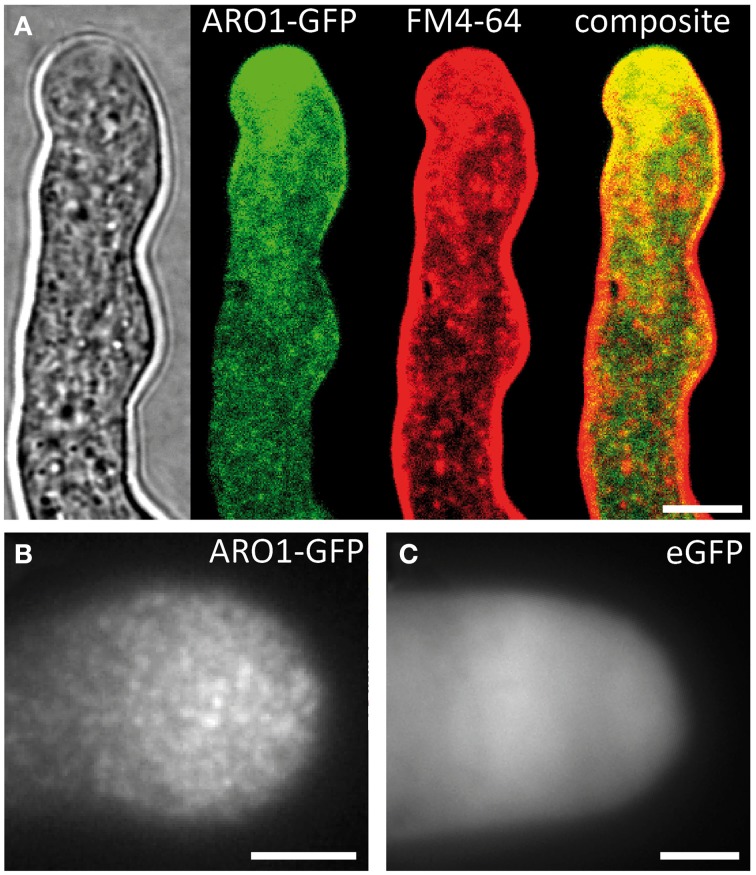
**ARO1-GFP localizes to vesicles at the pollen tube tip, accumulating in the inverted cone-shaped region**. (**A**) At the PT tip, ARO1-GFP predominantly accumulates in the vesicle-rich inverted cone-shaped region and partially co-localizes with FM4-64. No co-localization of ARO1-GFP and FM4-64-stained membrane compartments is detected in the subapical region of the PT. (**B**) TIRF microscopy reveals that ARO1-GFP signals appear as discrete punctate structures of approximately 0.2 μm in the PT tip. These punctate structures are not observed in PTs that express cytoplasmic GFP **(C)**. Scale bars: 5 μm.

In the growing PT the GFP fusion of Armadillo Repeat Only 1 (ARO1) accumulates in the vesicle-rich “clear zone” (Figure 3A; Supplemental Movie 2). We observed partial co-localization of ARO1-GFP fluorescence with FM4-64 in the “clear zone,” but almost no co-localization in the subapical part of the PT (Figure 3A). Spinning Disc confocal time-lapse imaging of growing PTs furthermore revealed that ARO1-GFP streams in a reverse fountain pattern (Supplemental Movie 2). The fact that ARO1-GFP accumulates in the PT tip in a brefeldin A-sensitive manner (Gebert et al., 2008) suggested that ARO1-GFP is associated to vesicles in the PT tip.

We performed TIRF microscopy to confirm the proposed vesicle-association of ARO1-GFP. By illuminating only a thin region of the PT tip, including the cytoplasmic zone immediately beneath the PT plasma membrane, we compared the fluorescent signals of ARO1-GFP with that of free GFP. As shown in Figure 3B, ARO1-GFP signals appeared as numerous dot-like structures with a size of approximately 200 nm in the PT tip. In contrast PTs expressing cytoplasmic GFP showed a homogenous fluorescence (Figure 3C).

### ARO1-GFP decorated vesicles peak at the future germination site during pollen activation

**Figure 4:**
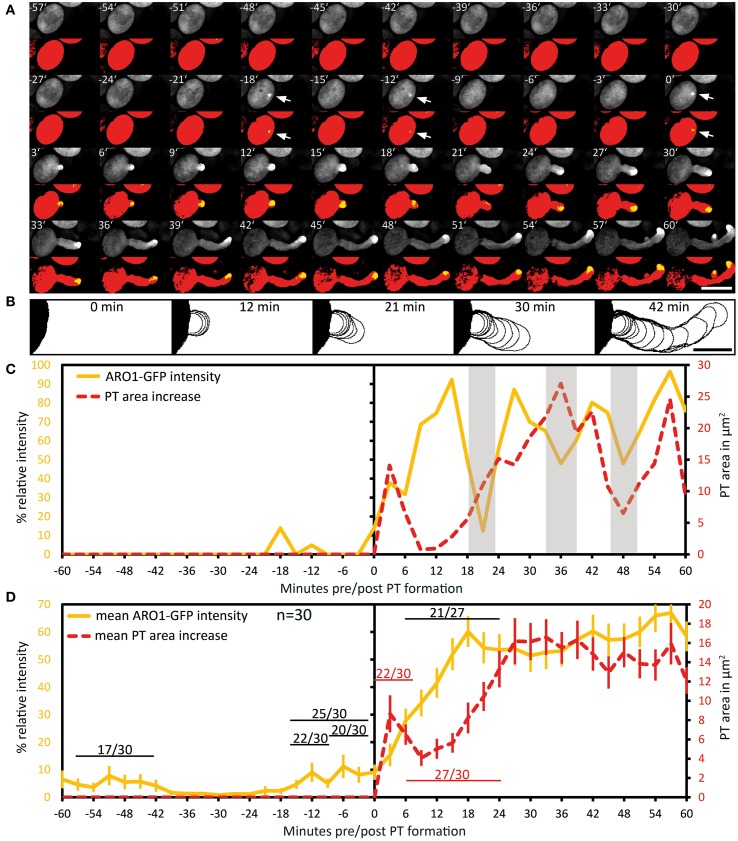
**Polarization of vesicle trafficking in activated pollen predetermines the site of pollen tube emergence**. Time series of fluorescence signal in germinating pollen expressing *P_*ARO*1_:ARO1-GFP*. Maximum intensity projected fluorescence raw signal and thresholded signals of a representative PT are shown in **(A)**. GFP fluorescence is shown in yellow (0.5% of highest intensities), and in red (70% of highest intensities) representing the ARO1-GFP maxima and the pollen cytoplasm. Outlines of PT shape for successive frames are drawn in **(B)**. The frame-wise increases in PT area over time and relative ARO1-GFP intensities for the PT in **(A)** are plotted in **(C)**. Gray shaded areas indicate phases of PT growth reorientation. Mean value ± 1 SE of frame-wise PT area increase and normalized ARO1-GFP signal maxima for 30 PTs are given in **(D)**. Numbers in **(D)** represent the frequency of an observed pattern. Time point 0′ indicates the last frame before germination. Arrows point to ARO1-GFP intensity maxima before germination. Scale bars: **(A)** 25 μm, **(B)** 10 μm.

We then investigated the subcellular localization and signal intensity changes of vesicle-associated ARO1-GFP before and during pollen germination using Spinning Disc microscopy (Figure 4A; Supplemental Movie 3). The respective outlines of the frame wise increase in PT area are shown in Figure 4B. The quantification of ARO1-GFP signal intensity in relation to the increase in PT area is given in Figure 4C.

Notably, we observed 18 and 12 min before pollen germination high intensity peaks of ARO1-GFP subjacent to the future site of PT outgrowth (arrows in Figure 4A; peaks in Figure 4C), resembling two knocks on the door. During the following bulging phase (0–12 min), ARO1-GFP steadily accumulated at the distal pole of the bulge (Figures 4A,C). High fluorescence intensities at the PT tip, shaped as inverted cone, were observed when the PT switched to rapid tip growth (24–60 min), with moderate downturns during short phases of tube growth re-orientation (30–36 min; 45–48 min), which also took place during the transition to rapid tip growth (18–24 min).

The quantitative analysis of 30 PTs is shown in Figure 4D. Sixty to thirty six minutes before germination, intensity peaks of ARO1-GFP appeared in 17 out of 30 pollen grains (Supplemental Movie 3). These intensity peaks, indicating rapid and local vesicle accumulation, were often but not always located near the future site of PT outgrowth. However, shortly before germination in 25 out of 30 pollen at least one high-intensity peak was detected subjacent to the future site of PT outgrowth (Figure 4D). 22 of 30 pollen grains showed one peak 12 to 9 min before germination at the future germination site, and in two thirds of observed pollen the ARO1-GFP high-intensity peak was recorded 6 to 3 min before germination. In 50% of the pollen two high-intensity peaks were visible (Supplemental Movie 3), while one third of pollen showed a single peak before germination. Frequencies and statistics of ARO1-GFP intensity peaks in pollen subjacent to the future site of PT outgrowth are shown in Figure S6.

Taken together, the temporary polar accumulation of ARO1-GFP decorated vesicles in activated pollen precedes pollen germination and marks the future site of PT outgrowth. Furthermore, we observed a strong increase of ARO1-GFP signal intensity after bulging, indicating the transition to rapid PT elongation (Figure 4D). Like observed for PTs expressing cytoplasmic GFP, almost all (27 of 30) ARO1-GFP expressing PTs showed an initial lag phase of growth after germination. During this lag phase, including bulging and transition phase, ARO1-GFP signal intensity strongly increased at the distal end of the bulge/dome-shaped tip in 21 of 27 PTs. Nine minutes after ARO1-GFP reached its maximum signal intensity at the tip of the tube, PT elongation rates reached their maxima, recognized by the rapid increase in PT area over time (Figure 4D). By contrast, during the first 9 min of the bulging phase 22 of 30 bulges substantially expanded while the accumulation of ARO1-GFP decorated vesicles at the distal end of the bulge was delayed, suggesting that bulging does not depend on pronounced vesicle trafficking to the very tip of the bulge.

### Patterns of abnormal PT growth correlate with deviating ARO1-GFP signals

**Figure 5:**
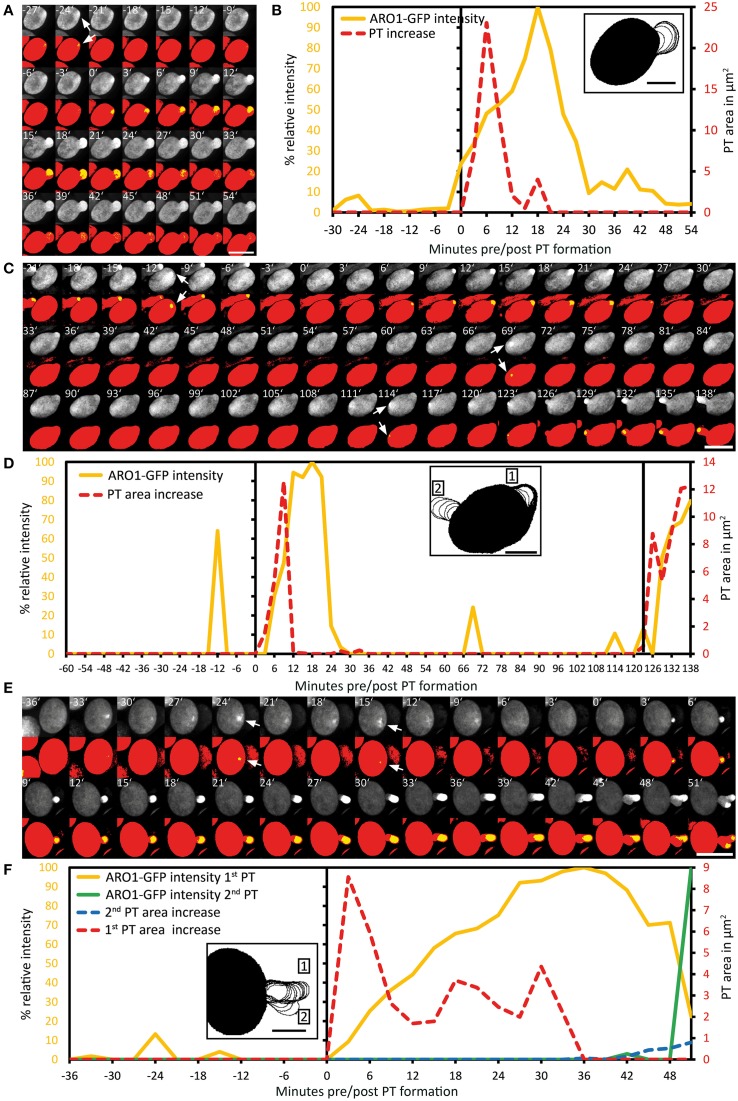
**Exceptional pollen germination events confirm the correlation between local vesicle accumulation and pollen tube emergence**. The breakdown of vesicle accumulation in the pollen tube bulge is accompanied with the failure to switch to rapid PT tip growth **(A**,**B)**. After unsuccessful transition to rapid PT tip growth, the pollen cell may also change the direction of polar vesicle trafficking, resulting in the establishment of a second germination site, as shown in **(C,D)**. A branching pollen tube is shown in **(E,F)**. After successful bulging the transition to rapid PT tip growth fails and a second growth site is selected, indicated by the accumulation of ARO1-GFP decorated vesicles in the tip of the PT branch. Maximum intensity projected fluorescence raw images and composite thresholded images are shown in **(A,C,E)**. GFP fluorescence is shown in yellow (0.5% of highest intensities) and in red (70% of highest intensities), representing the ARO1-GFP maxima and the pollen cytoplasm. Frame-wise PT area increase and relative ARO1-GFP intensity are shown in **(B,D,F)**, where insets show frame-wise overlaid PT shape outlines. Time point 0′ indicates the last frame before germination. Arrows point to ARO1-GFP intensity maxima before germination. Scale bars: **(A,C,E)** 25 μm, insets in **(B,D,F)** 10 μm.

Using our multi-well micro-germination setup a high number of pollen germinated and thus we were able to observe very rare events (less than 3.3%) of abnormal PT growth, such as bulging without subsequent elongation (Figures 5A,B), the initiation of a second PT from one grain (Figures 5C,D) or the branching of a PT (Figures 5E,F).

In the first case of a PT that did not switch to the phase of rapid PT elongation, a high intensity peak of ARO1-GFP signals appeared 24 min before germination at the future site of PT outgrowth (Figures 5A,B). During germination a roundish PT bulge was formed showing isodiametric expansion and constant increase in ARO1-GFP fluorescence intensity with a maximum 18 min after germination. However, during the following 12 min ARO1-GFP fluorescence rapidly decreased to only 10%, detected 30 min after germination. The decrease in ARO1-GFP signal intensity was accompanied with arrested PT growth (Figures 5A,B).

In one pollen grain a second tube was established during germination (Figures 5C,D). A sharp ARO1-GFP intensity maximum appeared subjacent to the future site of PT outgrowth, 12 min before pollen germination, the area of the bulge increased after pollen germination and ARO1-GFP accumulated in the bulge. However, 21 min after germination, ARO1-GFP fluorescence rapidly decreased and during the following 45 min neither the PT area increased, nor was any ARO1-GFP intensity maximum observed. Sixty nine minutes after first bulging, a new ARO1-GFP intensity maximum arose within the pollen grain, followed by another peak at the same site 45 min later. Nine minutes after the second ARO1-GFP fluorescence maximum the pollen grain started to germinate at this site. After bulging, ARO1-GFP steadily accumulated at the distal end of the second bulge and rapid PT elongation was successfully initiated (Figures 5C,D).

In the case of a branching PT (Figures 5E,F), two maxima of ARO1-GFP intensity occurred 24 and 15 min before pollen germination. During the following phase of bulging, ARO1-GFP signal intensity at the distal end of the bulge steadily increased and reached a maximum in the remarkable long transition phase, 36 min after germination. However, the switch to rapid PT growth did not occur at this site but a second growth site was selected, marked by ARO1-GFP signals appearing at the tip of the branching PT (Figure 5E; 42 min). During the following 6 min ARO1-GFP signal intensity at the first tube tip rapidly decreased while the PT branch expanded. Nine minutes after the PT initiated branching, another ARO1-GFP intensity maximum was detected in the new tip of the PT, while the signal in the old tip diminished (Figures 5E,F; 51 min).

### The actin cytoskeleton polarizes prior to germination and undergoes characteristic changes during PT growth

**Figure 6:**
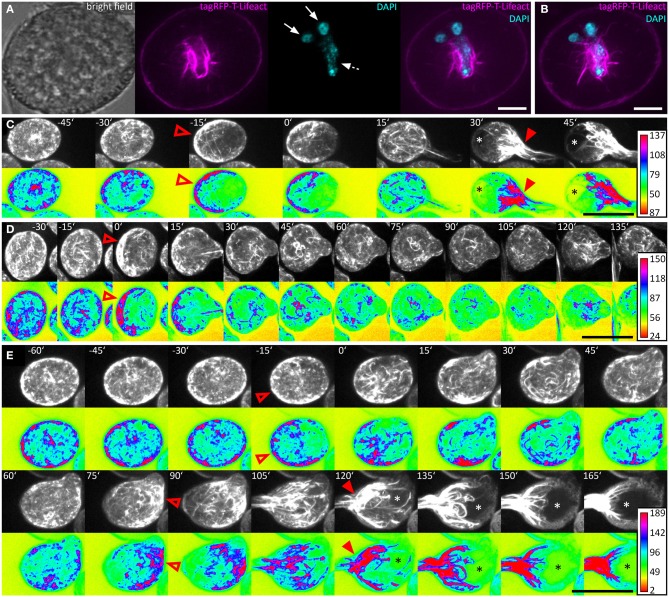
**The actin cytoskeleton undergoes characteristic changes during pollen germination**. Pollen expressing tagRFP-T-Lifeact was used for live-cell imaging of F-actin architecture and dynamics during pollen germination and growth. A single optical slice through a representative DAPI stained pollen grain is shown in **(A)**. Solid arrows mark the sperm cells and dashed arrows the vegetative nucleus. A composite maximum intensity projection of 19 optical slices is shown in **(B)**, the typical pattern of actin dynamics during pollen germinating is presented in **(C)**. A pronounced F-actin network accumulates at the periphery of the pollen vegetative cell, opposite to the future site of PT outgrowth (open arrowhead). Rapid PT tip growth is associated with vacuole formation (asterisk) and the massive appearance of parallel F-actin bundles extending from within the pollen grain, into the pollen tube (solid arrowheads). In **(D)** a PT that failed to switch to rapid PT tip growth after bulging is shown. The germination of a second PT after first unsuccessful bulging is shown in **(E)**. Maximum intensity projections of raw images and intensity based false-colored images are shown in **(C–E)** with respective calibration bars. Open red arrowheads indicate polar F-actin accumulation at the periphery of the pollen vegetative cell. Solid red arrowheads point at massively accumulating F-actin bundles that extend into the PT. Asterisks mark vacuoles. Time point 0′ indicates the last frame before germination. Scale bars: **(A)** 10 μm, **(B)** 5 μm **(C–E)** 25 μm.

We used the *ARO1* promoter to drive moderate expression of the tagRFP-T-Lifeact fusion protein in pollen. DAPI staining was performed to visualize the nuclei of the vegetative cell and the sperm cells in pollen grains. Immediately after pollen mounting, the pollen actin cytoskeleton was not distributed with any polarity and showed homogenous accumulation in the cell periphery and pronounced fluorescent signals around the vegetative nucleus (Figures 6A,B), which has also been reported for mature *Brassica napus* pollen (Hause et al., 1992; Gervais et al., 1994).

We germinated tagRFP-T-Lifeact expressing pollen in our micro-germination setup and observed that within half an hour before germination F-actin accumulated at the periphery of the pollen vegetative cell, opposite to the future site of PT outgrowth (Figures 6C–E). Almost all (35 of 38) pollen grains showed this pattern of F-actin polarization before germination.

Within 15 min after pollen germination, we observed an increase in longitudinal actin cable formation pointing toward the PT axis and partially reaching into the tube (Figure 6C). Very articulate actin reorganization appeared in all investigated PTs around 30 min after germination, when the PTs reached a mean size of 300 ± 17 μm^2^. The actin cytoskeleton assembled at the site of PT outgrowth forming prominent longitudinal F-actin bundles that reached from the pollen grain into the PT. The formation of a large vacuole opposite to the germination site was observed simultaneously with the prominent F-actin assembly near the site of PT outgrowth (Figure 6C). In later stages of PT growth, this dense assembly of F-actin cables was shifted into the PT (Supplemental Movie 4).

Again, we looked for exceptional germination scenarios and identified a PT that stopped growth after bulging (Figure 6D) and a PT that initiated a second tube from one pollen grain (Figure 6E). In the case of PT growth arrest after bulging, the F-actin polarized prior to germination at the pole opposite to the germination site and longitudinal actin cables reaching from the pollen grain into the PT bulge were present 15 min after germination. However after 30 min, the actin cytoskeleton started to depolarize and transition to rapid tip growth was not initiated (Figure 6D).

In the case of additional tube formation from one pollen grain, 15 min before germination the actin cytoskeleton accumulated at the periphery, opposite to the future site of PT outgrowth but after bulge formation, the F-actin almost completely depolarized until 60 min after the first germination (Figure 6E). F-actin repolarization was observed 75 min after the first germination event, opposing to the site where the second tube bulged later on. Fifteen to thirty minutes after the second F-actin polarization was observed at the periphery of the pollen vegetative cell, a second bulge was formed and underwent transition to rapid tip growth, showing all F-actin features of a normal growing PT.

### Sperm cell transport starts when the switch to rapid tip growth has taken place

**Figure 7:**
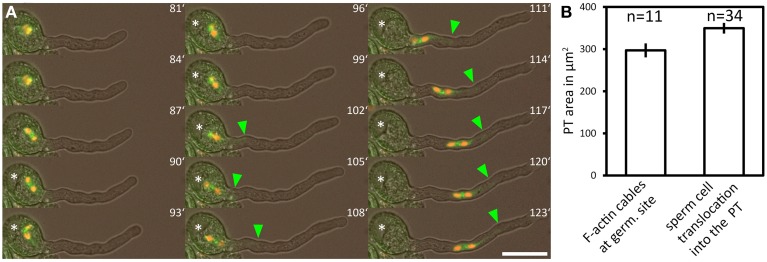
**The male germ unit is transported into the PT after the transition to rapid tip growth**. A marker line labeling both sperm nuclei (red fluorescence) and the sperm plasma membrane (green fluorescence) was used for time lapse imaging of pollen germination. **(A)** Green arrowhead points to the tip of the long sperm cell membrane extension physically associated to the vegetative cell nucleus, which has been transported into the pollen tube. Bar plots in **(B)** show average PT areas when F-actin bundles massively accumulate at the germination site (indication for rapid tip growth), compared to the mean PT area when the male germ unit is relocated into the PT. Scale bar in **(A)**: 25 μm.

We used a marker line showing RFP fluorescence in the sperm cell nuclei and GFP fluorescence in the sperm cell plasma membranes to investigate whether the two sperm cells are relocated into the PT at a distinct growth phase (Supplemental Movie 5). The GFP-labeled sperm cell membranes show that the two sperm cells are closely interlinked and that one long membrane extension connects one of the sperm cells to the nucleus of the vegetative cell (Figure 7), thereby forming a transport unit known as the male germ unit (MGU). In Arabidopsis, the entrance of the three MGU components into the PT follows a regular order, both in planta (Lalanne and Twell, 2002) and in *in vitro* germinated pollen (Zhou and Meier, 2014): the vegetative nucleus always precedes the sperm cells during entrance into the PT (Lalanne and Twell, 2002; own observations).

The tip of the GFP labeled long membrane extension of the leading sperm was used as a tracer for the position of the vegetative nucleus as it is hooked up to the vegetative nucleus. We defined the time point of MGU relocation into the PT when the tip of the sperm membrane extension became permanently visible outside the pollen grain (Figure 7A, green arrowhead). From 34 PTs we calculated a mean PT size of 350 ± 13 μm^2^ at the time point of MGU translocation into the PT (Figure 7B), indicating that the sperm cell transport into the PT does not occur at random but when the PT has reached a certain length and growth phase. Two processes associated with rapid PT tip growth, the formation of a large vacuole within the pollen grain and the accumulation of prominent F-actin cables at the base of the growing PT (Figure 7B), have already taken place when we detected the sperm membrane extension in the PT.

## Discussion

The PT is an attractive model for the analysis of tip growth mechanisms on the molecular and cellular level, especially in plant species amenable to forward genetic screens and with excellent genomic and bioinformatic resources such as *Arabidopsis thaliana*. Nevertheless, quantitative imaging of growing Arabidopsis PTs remained challenging, due to highly variable pollen germination rates in different experiments.

Here, we describe an inexpensive and easy mounting technique to simultaneously track germinating pollen from up to 12 genetically different plants. Our multi-well germination-slide with modified pollen germination medium yields high germination percentages and allows live cell imaging and subsequent quantitative image analysis of the whole process of Arabidopsis pollen activation, germination, and the establishment of polar tip growth.

By using our setup and pollen from different fluorescent marker lines we were able to precisely describe the kinetics of Arabidopsis pollen germination *in vitro*. We expressed tagRFP-T-Lifeact in pollen to investigate F-actin dynamics during pollen activation, germination and tube growth, as Lifeact has become the actin marker of choice in the PT (Qu et al., 2015). Vesicles within the pollen grain and the germinating PT were visualized by ARO1-GFP (Gebert et al., 2008). The accumulation of ARO1-GFP in the apical region of growing PTs and the rapid dissipation of this tip localization by brefeldin A treatment is reminiscent of YFP-RabA4d, an exocytotic vesicle marker of PTs (Lee et al., 2008; Szumlanski and Nielsen, 2009), and of GFP-Rab11b-tagged vesicles in tobacco PTs (de Graaf et al., 2005; Cheung and Wu, 2008). Transport vesicles in the tip of angiosperm PTs are known to follow a reverse fountain-streaming pattern (for review see Bove et al., 2008; Cheung and Wu, 2008; Chebli et al., 2013), as is the case for ARO1-GFP (Supplemental Movie 2). By TIRF microscopy, a method that has been successfully used to image secretory vesicles in *Picea meyeri* PTs (Wang et al., 2006), we were able to show that ARO1-GFP is associated to vesicles in the PT tip. The size of the punctate ARO1-GFP signals was approximately 200 nm in diameter, which is very close to the calculated size of 182 nm described for vesicles in Arabidopsis PTs (Ketelaar et al., 2008). Based on the vesicle-like appearance of ARO1-GFP in TIRF microscopy, its reverse fountain-streaming pattern and the BFA sensitive tip localization we conclude that ARO1-GFP shows a bona-fide vesicle association in the tip of growing PTs.

### Germinating arabidopsis pollen reveal characteristic tube morphologies and growth kinetics, accompanied with F-actin and vesicle polarization

**Figure 8:**
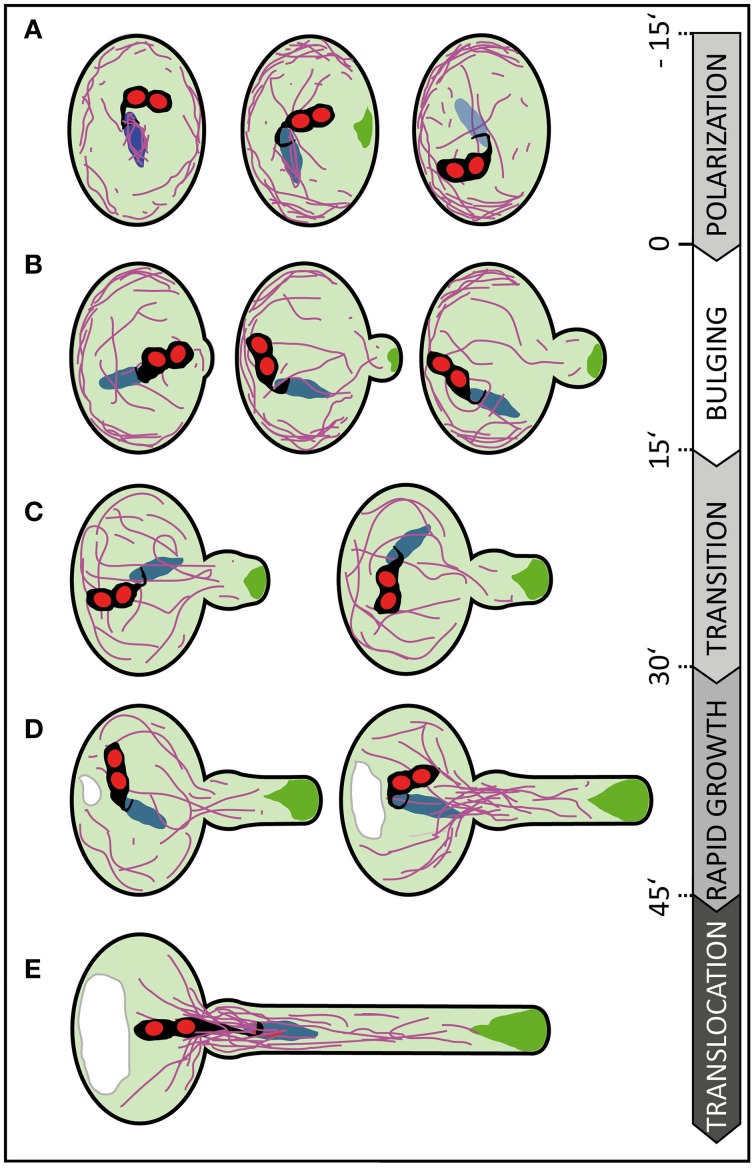
**Scheme summarizing subcellular changes observed during different phases of pollen germination and tube growth**. At least five distinct phases were recognized in our live cell imaging studies on *in vitro* germinating Arabidopsis pollen. **(A)** In early rehydrating pollen F-actin is uniformly distributed at the pollen cell cortex and forms prominent bundles around the vegetative nucleus. Polarization of the pollen grain is indicated by ARO1-GFP decorated vesicles, transiently accumulating subjacent to the future germination site approximately 3–20 min prior germination, and by F-actin accumulating at the cell periphery, in the half of the pollen vegetative cell opposite to the later germination site. **(B)** During the following bulging phase a local protuberance becomes visible, showing isodiametric expansion. ARO1-GFP decorated vesicles start to accumulate in the bulge and first longitudinal F-actin bundles extend from the grain into the bulge. **(C)** The transition phase is indicated when the bulge becomes slightly tubular-shaped. Transition to tip growth is accompanied by a strong accumulation of ARO1-GFP decorated vesicles in the shape of an inverted cone and by the reorganization of the actin cytoskeleton. The polar dense F-actin at the cell periphery of the pollen grain dissipates and long actin bundles, often oriented toward the emerging PT, arise. **(D)** During the subsequent phase of rapid tip growth the PT area increases significantly. A vacuole is formed in the pollen grain, across from the germination site and F-actin bundles start to extend from within the pollen grain into the pollen tube. The accumulation of ARO1-GFP-decorated vesicles in the very tip of the growing pollen tube is most pronounced. **(E)** The translocation phase is initiated when the MGU becomes transported into the growing pollen tube. Sperm cell translocation is preceded by the formation of massive parallel F-actin bundles at the germination site. The vacuole in the pollen grain rapidly enlarges. Objects are not to scale. Color code: purple lines, F-actin; green areas, ARO1-GFP; red areas, sperm cell nuclei; black areas surrounding sperm cell nuclei, sperm cell membranes and cytoplasm; blue area, vegetative cell nucleus. Numbers indicate approximate time points for each phase before or after germination in minutes.

Tip growing cells confine cellular expansion to a small area. The occurrence of a single growth site includes at least two distinct phases: the initiation of growth and the elongation phase (Geitmann, 2010). Our quantitative imaging of tube morphologies and growth kinetics enabled us to dissect the early events of germination and to define characteristic features associated with distinct phases (Figure 8). We observed successive phases of cell polarization before germination, bulge formation at the beginning of PT germination, the transition to polar growth and subsequent initiation of rapid tip growth.

The germination phase is characterized by an emerging PT that shows isodiametric expansion at the germination site. Some longer F-actin bundles become visible during that phase, extending from within the pollen grain into the bulge (Figure 8B). The growth rate of the expanding bulge, measured as increase in area over time, is rather slow during the first 15 min (16.7 ± 1.1 μm^2^), compared with later tube growth of 57.0 ± 4.0 μm^2^ at 45–60 min after germination. In our experimental setup this slower growth phase persists on average 30 min and includes the transition phase (Figure 8C) in which the bulge slightly elongates and adopts a dome-shaped form before switching to the phase of rapid tip growth. Notably, we observed that the bulge expands while the accumulation of ARO1-GFP associated vesicles at the distal end of the bulge is slightly delayed. The fact that the Arabidopsis PT starts forming a uniformly expanding bulge before vesicles massively accumulate at the future site of polar growth suggest that the bulging phase represents a rather turgor-driven deformation process, like assumed by Geitmann (2010). It furthermore indicates that the transition phase was preceded by the selection of a defined plasma membrane region for local exocytosis within the bulge. Thus, the burst of PTs soon after germination, especially observed in a number of pollen mutants such as *aro1-3* and *seth4* (Lalanne et al., 2004; Gebert et al., 2008), may be a turgor-driven event when the establishment of a local growth site was unsuccessful.

ARO1-GFP labeled vesicles heavily accumulate at the distal end of the bulge and finally adopt an inverted cone-like shape (Supplemental Movie 3). In elongating PTs the inverted cone-shaped zone at their apex is referred to as the “clear zone,” because this region is almost exclusively occupied by vesicles but lacks refracting starch containing amyloplasts (Hepler and Winship, 2015). The establishment of the vesicle-rich “clear zone” depends on acto-myosin-dependent long distance transport of vesicles toward the tip of the PT. This transport is mediated by F-actin cables, which are oriented parallel to the longitudinal axis of the PT and a cortical network of fine filaments located in the subapical region of the cell (Cai and Cresti, 2009; Chebli et al., 2013). Cortical actin filaments in the shank of angiosperm PTs are believed to be oriented with their barbed ends toward the apex, while the central actin bundles are thought to comprise filaments with the barbed ends pointing backwards (Chebli et al., 2013). The resulting reverse fountain-like cytoplasmic streaming observed in angiosperm PTs is likely to be involved in maintaining the “clear zone” by producing a constant shear between the anterograde and retrograde transport lanes, by which many of the vesicles, especially those near the surface of the inverted cone, will re-enter the tipward lanes and flow back to the apex (Hepler and Winship, 2015). Thus, the local accumulation and inverted cone-shaped appearance of ARO1-GFP labeled vesicles at the end of the bulging phase indicate that a distinct growth site has been selected, which also becomes apparent by the change in PT morphology and the increase in the median growth rate in the following transition phase (Figure 8C).

When rapid tip growth is initiated (Figure 8D), longitudinal actin cables extend from the pollen grain toward the apex of the PT and the volume of the vacuole opposite the germination site continuously increases. We observed the appearance of massive F-actin bundles near the germination site, extending from the pollen grain into the PT, when the PT area reached the average size of 300 μm^2^. Likewise, Rhodamine-phalloidin staining of *Pyrus communis* PTs showed articulate staining of actin at the PT base and of cables ranging into the tube (Tiwari and Polito, 1988). Notably, the male germ unit, comprising the two sperm cells associated to the vegetative cell nucleus, is transported from the pollen grain into the PT only when the PT completed its transition to rapid tip growth. In our experimental setup we detected the long membrane extension connecting the leading sperm cell to the vegetative nucleus in the PT when its area is 350 ± 13 μm^2^ (Figure 8E), which equals to a PT length of 63 ± 2.3 μm. Zhou and Meier (2014) determined a PT length of approximately 35 ± 10 μm when the vegetative cell nucleus permanently enters the PT. This difference may be attributed to a different experimental setup but also to the fact that Zhou and Meier (2014) used a marker line with a mCherry-labeled vegetative nucleus rather than labeled sperm nuclei and membranes as we did. While sperm cell nuclei are spheres, the vegetative nucleus is elongated and irregularly shaped and can reach a remarkable length (>20 μm) in the growing PT.

### New insights into arabidopsis pollen activation, provided by live imaging of vesicle dynamics and F-actin

Most pollen grains are metabolically quiescent and highly desiccated (Edlund et al., 2004). They need to attain a certain degree of hydration before they germinate, which will increase the turgor and transform the unpolar pollen grain to a highly polarized cell, a process termed pollen activation. In the past many studies were performed on morphological and ultrastructural changes in activated pollen revealing that, inter alia, the grain starts to organize its cytoskeleton and endoplasmic reticulum, and forms secretory vesicles (Raghavan, 1997 and references cited therein). Depending on the species examined, PTs either grow out of preformed germinal pores (apertures) or break directly through the exine wall, as is the case with Arabidopsis pollen grains. The presence of cytoplasmic vesicles subjacent to the aperture was detected by ultrastructural studies on pollen from *Lycopersicum peruvianum*, *Nicotiana alata* and *Narcissus pseudonarcissus* L. (Cresti et al., 1977, 1985; Heslop-Harrison and Heslop-Harrison, 1992). However, it is not yet clear how the pollen perceives external polarization signals and how they are transduced to select the site for tube emergence.

It was reported that the cytoplasmic Ca^2+^ concentration in Arabidopsis pollen increases at the potential germination site soon after hydration (Iwano et al., 2004) and that *in vitro* germination involves the formation of a “germination plaque” at the future site of tube emergence, containing cellulose, callose, pectin, and at least partly de-esterified pectin (Hoedemaekers et al., 2015). When we performed our live cell imaging on the dynamics of ARO-GFP1 labeled vesicles in hydrating pollen grains we observed the initial appearance of weak transient ARO1-GFP signals, arising at various areas of the pollen cell periphery. However, approximately 3–20 min before germination either one or two very strong fluorescent peaks of ARO1-GFP labeled vesicles appeared in the region where the PT protoplast will break through the exine, suggesting targeted vesicle secretion and probably local softening of the cell wall at this site. This would be in line with previous assumptions that vesicles filled with cell wall material and cell wall-modifying enzymes are directed toward the future emergence site to produce a local weak point at which the turgor-driven bulge formation is initiated afterwards (Krichevsky et al., 2007; Geitmann and Ortega, 2009; Cai et al., 2011).

The polarization of the actin cytoskeleton toward the site of tube emergence has been reported in activated *Pyrus communis* pollen (Tiwari and Polito, 1988) by using rhodamine-phalloidin labeling. Similar observations were made by TRITC-phalloidin staining for actin in hydrated *Narcissus pseudonarcissus* pollen (Heslop-Harrison and Heslop-Harrison, 1992). Notably, we did not observe a similar pattern of polarization in our live imaging setup with hydrating Arabidopsis pollen grains expressing tagRFP-T-Lifeact: F-actin mainly accumulated at the cell periphery opposite to the future germination site and no conspicuous polarization toward the site of tube emergence was observed before PT bulging. We assume that the spatial configuration of actin arrays at the periphery of the other half of the activated Arabidopsis pollen grain may form a mechanical counter-bearing for the turgor-driven PT bulging.

Species-dependent variations in F-actin polarization during pollen grain activation would be conceivable, on the other hand previous reports using actin-binding proteins or their actin-binding domains have shown that each F-actin marker produces a different labeling pattern (Thomas et al., 2006; Wilsen et al., 2006; Cheung et al., 2008). However, the distribution of the actin cytoskeleton in hydrating Arabidopsis pollen grains by fluorescent phalloidin has, to our knowledge, not been investigated in detail and will be difficult to interpret without having any information about the cellular dynamics before and after the moment of fixation.

## Conclusions

Our live imaging studies on germinating Arabidopsis PTs using the described mounting technique revealed characteristic growth phases and kinetics, together with specific spatiotemporal changes in vesicle transport and actin cytoskeletal organization. The method presented here allows the phenotypic assessment of larger numbers of *in vitro* germinating Arabidopsis pollen from wild type and mutant plants by live imaging. It facilitates the analyses of morphological alterations and growth kinetics, and the identification and subcellular localization of players contributing to cell polarity formation and growth site selection in germinating pollen.

## Author contributions

FV and SS designed the experiments, SS directed the project. Generation of constructs and transgenic plants, single- and multi-well micro-germination setup establishment, Spinning Disc microscopy, quantitative image analysis and statistics was carried out by FV. TIRF microscopy was performed by SK. FV wrote the manuscript with input from SK and SS.

### Conflict of interest statement

The authors declare that the research was conducted in the absence of any commercial or financial relationships that could be construed as a potential conflict of interest.

## Supplementary material

Supplemental Movie 1 **Time lapse imaging of pollen germinating in the multi-well micro-germination slide**. Imaging of ARO1-GFP expressing pollen in an exemplary 10 well micro-germination setup was performed over 6 h. Note that position B1 was readjusted after 1 h PT growth. Time stamper shows hours and minutes. Scale bar: 50 μm.

Supplemental Movie 2 **Vesicle-associated ARO1-GFP is enriched in the inverted cone-shaped “clear zone” of the pollen tube tip and shows reverse fountain streaming pattern**. Time lapse Spinning Disc confocal imaging of ARO1-GFP at the PT tip. Royal LUT was used to highlight increasing signal intensities (low to high: blue to yellow to red to white). Time stamper shows minutes and seconds. Scale bar: 5 μm.

Supplemental Movie 3 **Polar vesicle accumulation during pollen germination and PT tip growth, indicated by ARO1-GFP intensity changes**. Time lapse imaging of a representative pollen expressing ARO1-GFP before germination, during bulging and PT growth. PT outlines are drawn in cyan, ARO1-GFP low-intensity threshold in red (70% highest intensities) and signal maxima in yellow (0.5% highest intensities). White arrowheads point to ARO1-GFP maxima in the ungerminated pollen grain, green arrows indicate ARO1-GFP maxima after germination. Time stamper shows minutes pre/post germination. Scale bar: 10 μm.

Supplemental Movie 4 **F-actin dynamics during pollen germination and PT tip growth**. Time lapse imaging of tagRFP-T-Lifeact expressing pollen before germination and during PT growth. Solid arrowheads highlight F-actin assembly at the periphery of the pollen cell, opposite to the later germination site. Empty arrowheads point at the massive accumulation of F-actin bundles at the PT base, during rapid PT growth. Time stamper shows minutes pre/post germination. Scale bar: 15 μm.

Supplemental Movie 5 **Sperm cell translocation into the growing PT**. Time lapse imaging of germinating pollen with fluorescently labeled sperm cell nuclei and plasma membranes. Solid arrowheads highlight the sperm plasma membrane protrusion that connects one sperm cell to the vegetative cell nucleus. Time stamper shows minutes pre/post germination. Scale bar: 15 μm.

## Abbreviations

ARO: Armadillo Repeat Only
epiBL: 24-epibrassinolide
F-actin: filamentous actin
GFP: green fluorescent protein
PGM: pollen germination medium
PT: pollen tube
RFP: red fluorescent protein
TIRF: total internal reflection fluorescence.

## References

[B1] Bibikova T. Gilroy S. (2003). Root hair development. J. Plant Growth Regul. 21, 383–415 10.1007/s00344-003-0007-x

[B2] Boavida L. C. McCormick S. (2007). Temperature as a determinant factor for increased and reproducible *in vitro* pollen germination in *Arabidopsis thaliana*. Plant J. 52, 570–582. 10.1111/j.1365-313X.2007.03248.x 17764500

[B3] Bou Daher F. Chebli Y. Geitmann A. (2009). Optimization of conditions for germination of cold-stored *Arabidopsis thaliana* pollen. Plant Cell Rep. 28, 347–357. 10.1007/s00299-008-0647-1 19050898

[B4] Bove J. Vaillancourt B. Kroeger J. Hepler P. K. Wiseman P. W. Geitmann A. (2008). Magnitude and direction of vesicle dynamics in growing pollen tubes using spatiotemporal image correlation spectroscopy and fluorescence recovery after photobleaching. Plant Physiol. 147, 1646–1658. 10.1104/pp.108.120212 18508956 PMC2492615

[B5] Cai G. Cresti M. (2009). Organelle motility in the pollen tube: a tale of 20 years. J. Exp. Bot. 60, 495–508. 10.1093/jxb/ern321 19112169

[B6] Cai G. Faleri C. Del Casino C. Emons A. M. Cresti M. (2011). Distribution of callose synthase, cellulose synthase, and sucrose synthase in tobacco pollen tube is controlled in dissimilar ways by actin filaments and microtubules. Plant Physiol. 155, 1169–1190. 10.1104/pp.110.171371 21205616 PMC3046577

[B7] Campanoni P. Blatt M. R. (2007). Membrane trafficking and polar growth in root hairs and pollen tubes. J. Exp. Bot. 58, 65–74. 10.1093/jxb/erl059 16873451

[B8] Chebli Y. Kroeger J. Geitmann A. (2013). Transport logistics in pollen tubes. Mol. Plant 6, 1037–1052. 10.1093/mp/sst073 23686949

[B9] Cheung A. Y. Boavida L. C. Aggarwal M. Wu H. M. Feijo J. A. (2010). The pollen tube journey in the pistil and imaging the *in vivo* process by two-photon microscopy. J. Exp. Bot. 61, 1907–1915. 10.1093/jxb/erq062 20363865

[B10] Cheung A. Y. Duan Q. H. Costa S. S. de Graaf B. H. Di Stilio V. S. Feijo J.. (2008). The dynamic pollen tube cytoskeleton: live cell studies using actin-binding and microtubule-binding reporter proteins. Mol. Plant 1, 686–702. 10.1093/mp/ssn026 19825573

[B11] Cheung A. Y. Wu H. M. (2008). Structural and signaling networks for the polar cell growth machinery in pollen tubes. Annu. Rev. Plant Biol. 59, 547–572. 10.1146/annurev.arplant.59.032607.092921 18444907

[B12] Clough S. J. Bent A. F. (1998). Floral dip: a simplified method for Agrobacterium-mediated transformation of *Arabidopsis thaliana*. Plant J. 16, 735–743. 10.1046/j.1365-313x.1998.00343.x 10069079

[B13] Cresti M. Ciamolini F. Mulcahy D. L. M. Mulcahy G. (1985). Ultrastructure of *Nicotiana alata* pollen, its germination and early tube formation. Am. J. Bot. 72, 719–727 10.2307/2443685

[B14] Cresti M. Pacini E. Ciampolini F. Sarfatti G. (1977). Germination and early tube development *in vitro* of *Lycopersicum peruvianum* pollen: ultrastructural features. Planta 136, 239–247. 10.1007/BF00385991 24420397

[B15] de Graaf B. H. Cheung A. Y. Andreyeva T. Levasseur K. Kieliszewski M. Wu H. M. (2005). Rab11 GTPase-regulated membrane trafficking is crucial for tip-focused pollen tube growth in tobacco. Plant Cell 17, 2564–2579. 10.1105/tpc.105.033183 16100336 PMC1197435

[B16] Denninger P. Bleckmann A. Lausser A. Vogler F. Ott T. Ehrhardt D. W.. (2014). Male-female communication triggers calcium signatures during fertilization in Arabidopsis. Nat. Commun. 5, 4645. 10.1038/ncomms5645 25145880 PMC4143946

[B17] Edlund A. F. Swanson R. Preuss D. (2004). Pollen and stigma structure and function: the role of diversity in pollination. Plant Cell 16, S84–S97. 10.1105/tpc.015800 15075396 PMC2643401

[B18] Feijó J. A. Moreno N. (2004). Imaging plant cells by two-photon excitation. Protoplasma 223, 1–32. 10.1007/s00709-003-0026-2 15004740

[B19] Gebert M. Dresselhaus T. Sprunck S. (2008). F-actin organization and pollen tube tip growth in Arabidopsis are dependent on the gametophyte-specific Armadillo repeat protein ARO1. Plant Cell 20, 2798–2814. 10.1105/tpc.108.061028 18931021 PMC2590741

[B20] Geitmann A. (2010). How to shape a cylinder: pollen tube as a model system for the generation of complex cellular geometry. Sex. Plant Reprod. 23, 63–71. 10.1007/s00497-009-0121-4 20165964

[B21] Geitmann A. Ortega J. K. (2009). Mechanics and modeling of plant cell growth. Trends Plant Sci. 14, 467–478. 10.1016/j.tplants.2009.07.006 19717328

[B22] Gervais C. Simmonds D. H. Newcomb W. (1994). Actin microfilament organization during pollen development of *Brassica napus* cv. Topas. Protoplasma 183, 67–76 10.1007/BF01276814

[B23] Guan Y. Guo J. Li H. Yang Z. (2013). Signaling in pollen tube growth: crosstalk, feedback, and missing links. Mol. Plant 6, 1053–1064. 10.1093/mp/sst070 23873928 PMC3842152

[B24] Hause G. Hause B. Van Lammeren A. A. M. (1992). Microtubular and actin-filament configurations during microspore and pollen development in *Brassica napus* L. cv. Topas. Can. J. Bot. 70, 1369–1376 10.1139/b92-172

[B25] Hepler P. K. Winship L. J. (2015). The pollen tube clear zone: clues to the mechanism of polarized growth. J. Integr. Plant Biol. 57, 79–92. 10.1111/jipb.12315 25431342

[B26] Heslop-Harrison Y. Heslop-Harrison J. (1992). Germination of monocolpate angiosperm pollen: evolution of the actin cytoskeleton and wall during hydration, activation and tube emergence. Annals Bot. 69, 385–394.

[B27] Hoedemaekers K. Derksen J. Hoogstrate S. W. Wolters-Arts M. Oh S.-A. Twell D.. (2015). BURSTING POLLEN is required to organize the pollen germination plaque and pollen tube tip in *Arabidopsis thaliana*. New Phytol. 206, 255–267. 10.1111/nph.13200 25442716

[B28] Iwano M. Shiba H. Miwa T. Che F.-S. Takayama S. Nagai T.. (2004). Ca^2+^ dynamics in a pollen grain and papilla cell during pollination of Arabidopsis. Plant Physiol. 136, 3562–3571. 10.1104/pp.104.046961 15489279 PMC527155

[B30] Johnson-Brousseau S. A. McCormick S. (2004). A compendium of methods useful for characterizing Arabidopsis pollen mutants and gametophytically-expressed genes. Plant J. 39, 761–775. 10.1111/j.1365-313X.2004.02147.x 15315637

[B31] Karimi M. Inzé D. Depicker A. (2002). GATEWAY™ vectors for Agrobacterium-mediated plant transformation. Trends Plant Sci. 7, 193–195. 10.1016/S1360-1385(02)02251-3 11992820

[B32] Ketelaar T. Galway M. E. Mulder B. M. Emons A. M. (2008). Rates of exocytosis and endocytosis in Arabidopsis root hairs and pollen tubes. J. Microsc. 231, 265–273. 10.1111/j.1365-2818.2008.02031.x 18778424

[B33] Kost B. (2008). Spatial control of Rho (Rac-Rop) signaling in tip-growing plant cells. Trends Cell Biol. 18, 119–127. 10.1016/j.tcb.2008.01.003 18280158

[B34] Krichevsky A. Kozlovsky S. V. Tian G. W. Chen M. H. Zaltsman A. Citovsky V. (2007). How pollen tubes grow. Dev. Biol. 303, 405–420. 10.1016/j.ydbio.2006.12.003 17214979

[B35] Lalanne E. Michaelidis C. Moore J. M. Gagliano W. Johnson A. Patel R.. (2004). Analysis of transposon insertion mutants highlights the diversity of mechanisms underlying male progamic development in Arabidopsis. Genetics 167, 1975–1986. 10.1534/genetics.104.030270 15342534 PMC1471024

[B36] Lalanne E. Twell D. (2002). Genetic control of male germ unit organization in Arabidopsis. Plant Physiol. 129, 865–875. 10.1104/pp.003301 12068125 PMC161707

[B37] Lee Y. J. Szumlanski A. Nielsen E. Yang Z. (2008). Rho-GTPase–dependent filamentous actin dynamics coordinate vesicle targeting and exocytosis during tip growth. J. Cell Biol. 181, 1155–1168. 10.1083/jcb.200801086 18591430 PMC2442199

[B38] Lee Y. J. Yang Z. (2008). Tip growth: signaling in the apical dome. Curr. Opin. Plant Biol. 11, 662–671. 10.1016/j.pbi.2008.10.002 18977167 PMC2613292

[B39] Mascarenhas J. P. (1993). Molecular mechanisms of pollen tube growth and differentiation. Plant Cell 5, 1303–1314. 10.1105/tpc.5.10.1303 12271030 PMC160363

[B40] McCue A. D. Cresti M. Feijó J. A. Slotkin R. K. (2011). Cytoplasmic connection of sperm cells to the pollen vegetative cell nucleus: potential roles of the male germ unit revisited. J. Exp. Bot. 62, 1621–1631. 10.1093/jxb/err032 21357775

[B41] Müller M. Schmidt W. (2004). Environmentally induced plasticity of root hair development in Arabidopsis. Plant Physiol. 134, 409–419. 10.1104/pp.103.029066 14730071 PMC371035

[B42] Parker J. S. Cavell A. C. Dolan L. Roberts K. Grierson C. S. (2000). Genetic interactions during root hair morphogenesis in Arabidopsis. Plant Cell 12, 1961–1974. 10.1105/tpc.12.10.1961 11041890 PMC149133

[B43] Qin Y. Yang Z. (2011). Rapid tip growth: insights from pollen tubes. Semin. Cell Dev. Biol. 22, 816–824. 10.1016/j.semcdb.2011.06.004 21729760 PMC3210868

[B44] Qu X. Jiang Y. Chang M. Liu X. Zhang R. Huang S. (2015). Organization and regulation of the actin cytoskeleton in the pollen tube. Front. Plant Sci. 5:786. 10.3389/fpls.2014.00786 25620974 PMC4287052

[B45] Raghavan V. (1997). Molecular Embryology of Flowering Plants. Cambridge: Cambridge University Press 10.1017/CBO9780511574528

[B46] Riedl J. Crevenna A. H. Kessenbrock K. Yu J. H. Neukirchen D. Bista M.. (2008). Lifeact: a versatile marker to visualize F-actin. Nat. Methods 5, 605–607. 10.1038/nmeth.1220 18536722 PMC2814344

[B47] Rodriguez-Enriquez M. J. Mehdi S. Dickinson H. G. Grant-Downton R. T. (2013). A novel method for efficient *in vitro* germination and tube growth of *Arabidopsis thaliana* pollen. New Phytol. 197, 668–679. 10.1111/nph.12037 23173941

[B29] Šamaj J. Muller J. Beck M. Bohm N. Menzel D. (2006). Vesicular trafficking, cytoskeleton and signalling in root hairs and pollen tubes. Trends Plant Sci. 11, 594–600. 10.1016/j.tplants.2006.10.002 17092761

[B48] Sanati Nezhad A. Packirisamy M. Geitmann A. (2014). Dynamic, high precision targeting of growth modulating agents is able to trigger pollen tube growth reorientation. Plant J. 80, 185–195. 10.1111/tpj.12613 25041411

[B49] Schiefelbein J. W. (2000). Constructing a plant cell. The genetic control of root hair development. Plant Physiol. 124, 1525–1531. 10.1104/pp.124.4.1525 11115870 PMC1539308

[B50] Schiefelbein J. W. Somerville C. (1990). Genetic control of root hair development in *Arabidopsis thaliana*. Plant Cell 2, 235–243. 10.1105/tpc.2.3.235 12354956 PMC159880

[B51] Smyth D. R. Bowman J. L. Meyerowitz E. M. (1990). Early flower development in Arabidopsis. Plant Cell 2, 755–767. 10.1105/tpc.2.8.755 2152125 PMC159928

[B52] Sprunck S. Rademacher S. Vogler F. Gheyselinck J. Grossniklaus U. Dresselhaus T. (2012). Egg cell–secreted EC1 triggers sperm cell activation during double fertilization. Science 338, 1093–1097. 10.1126/science.1223944 23180860

[B53] Steinhorst L. Kudla J. (2013). Calcium - a central regulator of pollen germination and tube growth. Biochim. Biophys. Acta 1833, 1573–1581. 10.1016/j.bbamcr.2012.10.009 23072967

[B54] Szumlanski A. L. Nielsen E. (2009). The Rab GTPase RabA4d regulates pollen tube tip growth in *Arabidopsis thaliana*. Plant Cell 21, 526–544. 10.1105/tpc.108.060277 19208902 PMC2660625

[B55] Taylor L. P. Hepler P. K. (1997). Pollen germination and tube growth. Annu. Rev. Plant Phys. 48, 461–491 10.1146/annurev.arplant.48.1.461 15012271

[B56] Thomas C. Hoffmann C. Dieterle M. Van Troys M. Ampe C. Steinmetz A. (2006). Tobacco WLIM1 is a novel F-actin binding protein involved in actin cytoskeleton remodeling. Plant Cell 18, 2194–2206. 10.1105/tpc.106.040956 16905656 PMC1560925

[B57] Tiwari S. Polito V. (1988). Organization of the cytoskeleton in pollen tubes of *Pyrus communis*: a study employing conventional and freeze-substitution electron microscopy, immunofluorescence, and rhodamine-phalloidin. Protoplasma 147, 100–112 10.1007/BF01403337

[B58] Twell D. Yamaguchi J. McCormick S. (1990). Pollen-specific gene expression in transgenic plants: coordinate regulation of two different tomato gene promoters during microsporogenesis. Development 109, 705–713. 2401221 10.1242/dev.109.3.705

[B59] Vogler F. Schmalzl C. Englhart M. Bircheneder M. Sprunck S. (2014). Brassinosteroids promote Arabidopsis pollen germination and growth. Plant Reprod. 27, 153–167. 10.1007/s00497-014-0247-x 25077683

[B60] Wang X. Teng Y. Wang Q. Li X. Sheng X. Zheng M.. (2006). Imaging of dynamic secretory vesicles in living pollen tubes of *Picea meyeri* using evanescent wave microscopy. Plant Physiol. 141, 1591–1603. 10.1104/pp.106.080168 16798949 PMC1533916

[B61] Wilsen K. Lovy-Wheeler A. Voigt B. Menzel D. Kunkel J. Hepler P. (2006). Imaging the actin cytoskeleton in growing pollen tubes. Sex. Plant Reprod. 19, 51–62 10.1007/s00497-006-0021-9

[B62] Zhou X. Meier I. (2014). Efficient plant male fertility depends on vegetative nuclear movement mediated by two families of plant outer nuclear membrane proteins. Proc. Natl. Acad. Sci. U.S.A. 111, 11900–11905. 10.1073/pnas.1323104111 25074908 PMC4136564

