# Supplementary material for: Knockin' on pollen's door: live cell imaging of early polarization events in germinating Arabidopsis pollen
Source: Front Plant Sci. 2015 Apr 21;6:246. doi: 10.3389/fpls.2015.00246 (PMC4404733; doi:10.3389/fpls.2015.00246)
Supplement: Supplementary file 8 [file Image3.PDF]

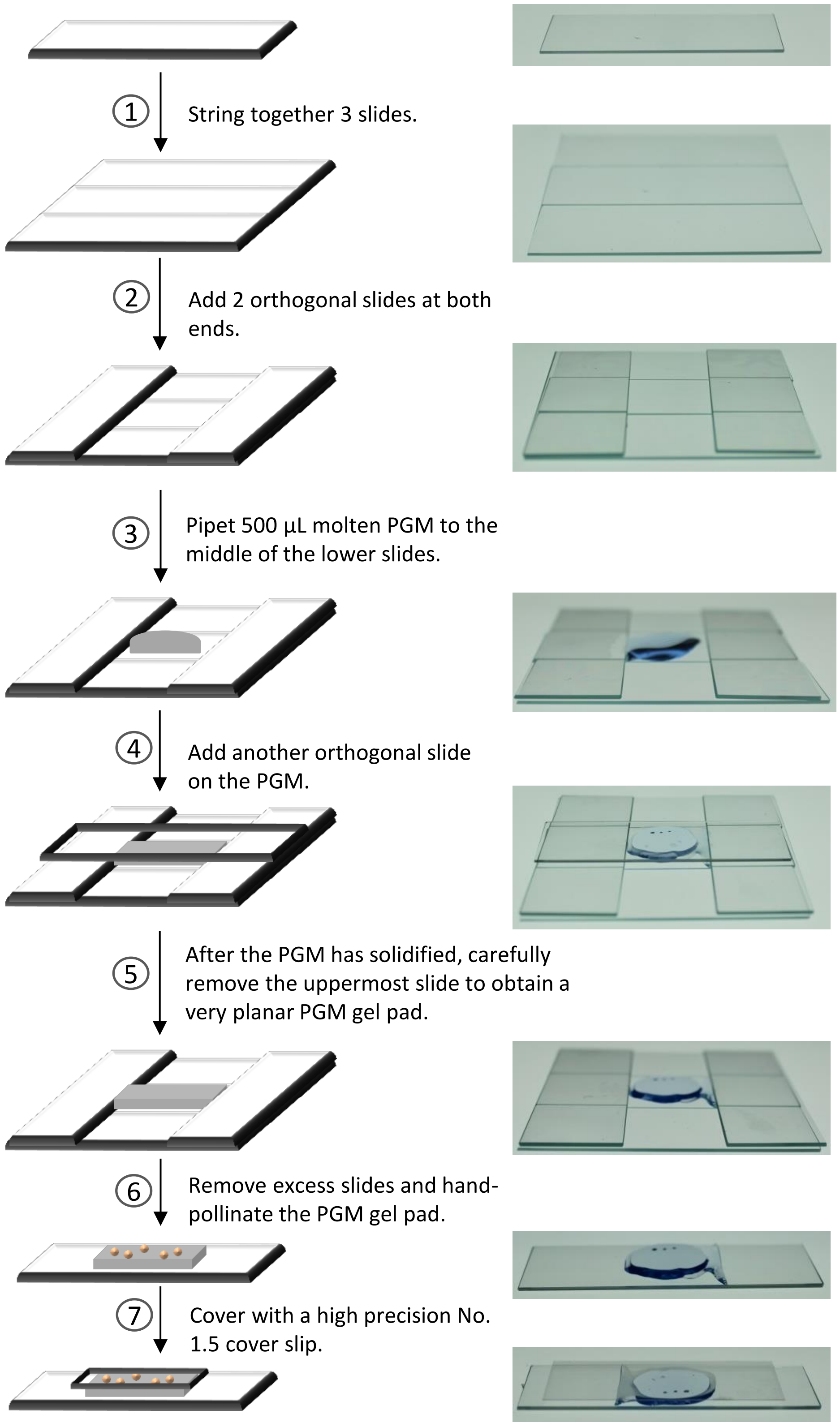

Figure S3: Preparing a germination slide for TIRF microscopy
Mounting scheme as described in Material & Methods section. In the pictures on the right hand side Bromphenol blue was added to the PGM for a better visualization.
