# Supplementary material for: Knockin' on pollen's door: live cell imaging of early polarization events in germinating Arabidopsis pollen
Source: Front Plant Sci. 2015 Apr 21;6:246. doi: 10.3389/fpls.2015.00246 (PMC4404733; doi:10.3389/fpls.2015.00246)
Supplement: Supplementary file 9 [file Image4.PDF]

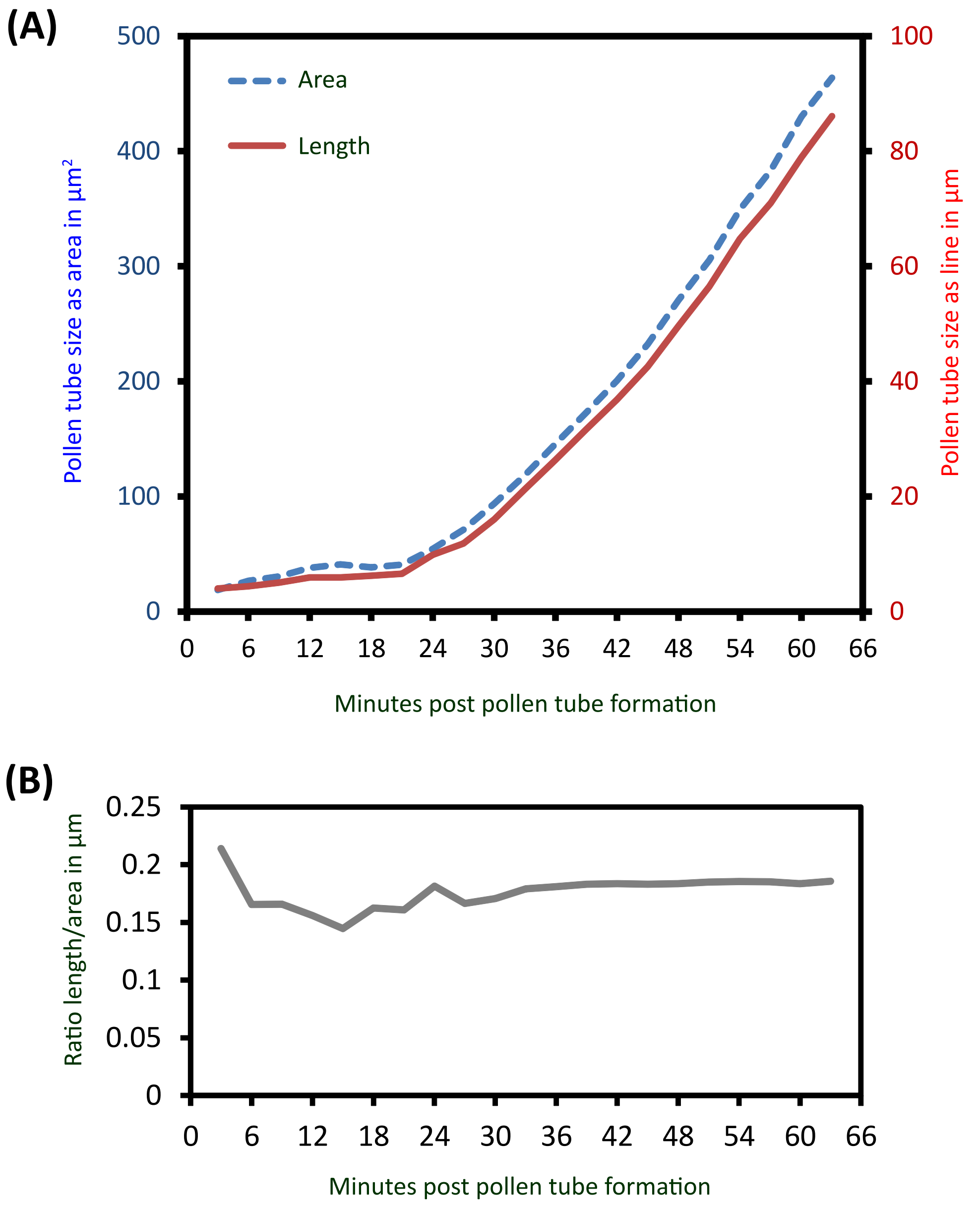

Figure S4: Pollen tube growth kinetics, measured as frame-wise increase in pollen tube area and in pollen tube length

The pollen tube shown in Figure 2A was used to compare its increase in area (μm^2) over time with increasing tube length (µm), measured along a segmented line from the germination site to the PT tip **(A)**. The ratio of length and area over time for this PT is shown in **(B)**. Note that especially during the first 30 minutes after pollen germination (bulging and transition phase), subtle differences are visible between the two methods, with a slightly better resolution in PT growth dynamics when measuring PT areas. The ratio length/area furthermore reveals that the PT area can be divided by 0.18 μm^{-1} which is the mean of all values shown in **(B)**, to yield the approximate length of a PT in µm.
