# Supplementary material for: Knockin' on pollen's door: live cell imaging of early polarization events in germinating Arabidopsis pollen
Source: Front Plant Sci. 2015 Apr 21;6:246. doi: 10.3389/fpls.2015.00246 (PMC4404733; doi:10.3389/fpls.2015.00246)
Supplement: Supplementary file 10 [file Image5.PDF]

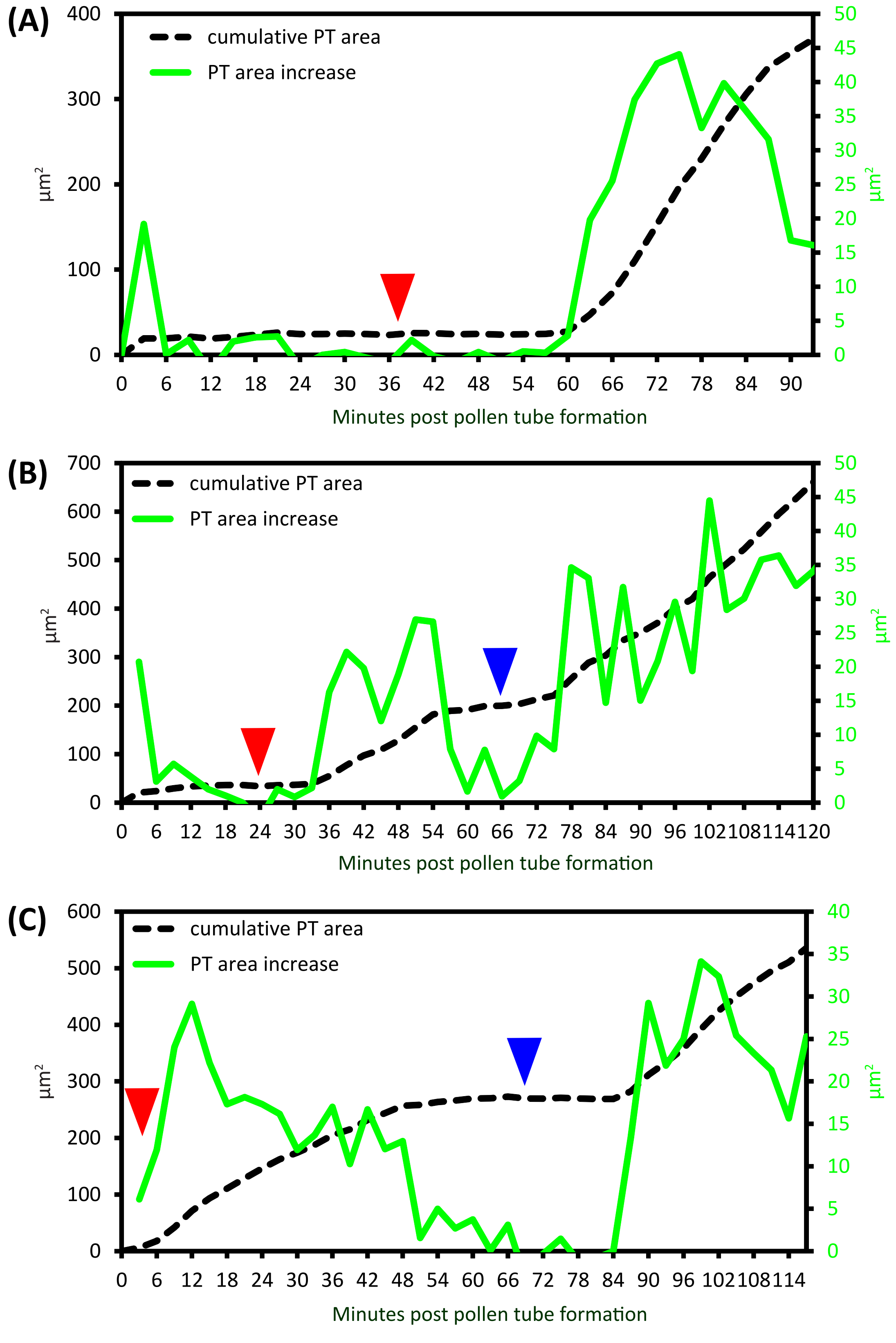

Figure S5: Deviating lag phases observed during in vitro pollen tube growth
PTs exhibiting varying lag phases. **(A)** PT with extended bulging phase. **(B)** and **(C)** show a second lag phase that can eventually be observed during rapid PT growth. Note that in **(C)** this second lag phase is very pronounced. Red arrowheads mark the first, blue arrowheads the second lag phase.
