# Supplementary material for: Knockin' on pollen's door: live cell imaging of early polarization events in germinating Arabidopsis pollen
Source: Front Plant Sci. 2015 Apr 21;6:246. doi: 10.3389/fpls.2015.00246 (PMC4404733; doi:10.3389/fpls.2015.00246)
Supplement: Supplementary file 11 [file Image6.PDF]

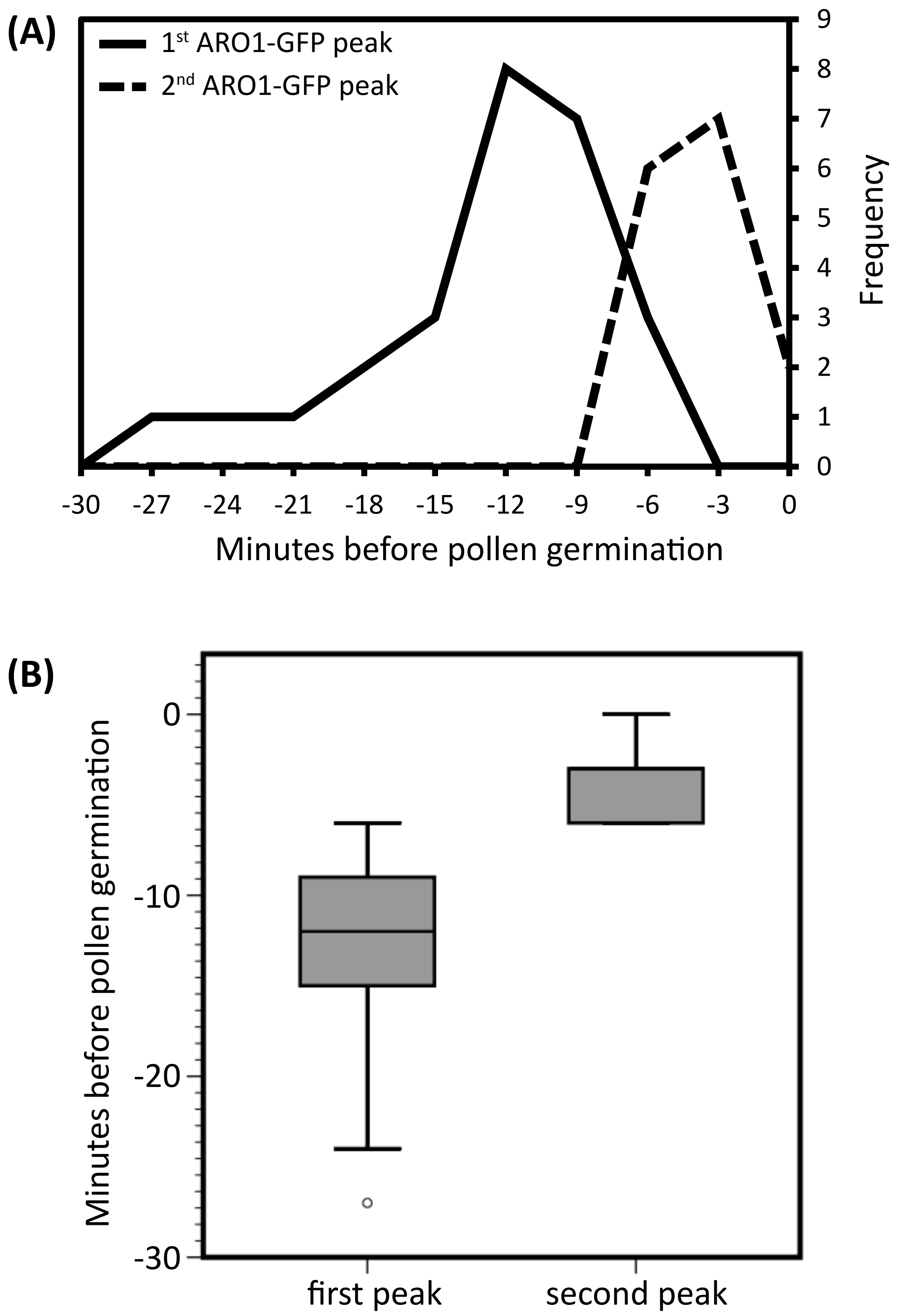

Figure S6: Frequencies and time points of ARO1-GFP intensity peaks in pollen grains before germination

In 25 of 30 pollen investigated, ARO1-GFP showed one to two signal intensity peaks within 30 minutes before germination, indicating rapid and local accumulation of ARO1-GFP decorated vesicles at the periphery of the pollen grain protoplast. The frequencies of observed intensity maxima are shown in **(A)** and corresponding boxplots in **(B)**.
